# Experimental and Molecular Simulation Study of Methyl Orange Adsorption on ZIF-67

**DOI:** 10.3390/ijms27177578

**Published:** 2026-08-24

**Authors:** Lili Gao, Kalampyr Bexeitova, Inabat Sapargali, Kenes Kudaibergenov, Alzhan Baimenov, Tiancheng You, Seitkhan Azat, Jechan Lee

**Affiliations:** 1 Laboratory of Engineering Profile, Satbayev University, Satbayev Str. 22, Almaty 050000, Kazakhstan; gao.l@stud.satbayev.university (L.G.); kalampyr.bexeitovas@gmail.com (K.B.); sapargaliinabat60@gmail.com (I.S.); k.kudaibergenov@satbayev.university (K.K.); alzhan.baimenov@satbayev.university (A.B.); 2 School of Energy and Constructional Engineering, Shandong Huayu University of Technology, Dezhou 253000, China; 3 Powder Metallurgy Research Institute, Central South University, Changsha 410083, China; 4 Department of Global Smart City, School of Civil, Architectural Engineering, and Landscape Architecture, Sungkyunkwan University, Suwon 16419, Republic of Korea

**Keywords:** ZIF-67, adsorption performance, kinetic modeling, anionic dye adsorption

## Abstract

Efficient removal of organic dye pollutants remains challenging owing to the limited adsorption capacity and poor reusability of most conventional adsorbents. Herein, rhombic-dodecahedral zeolitic imidazolate framework-67 (ZIF-67) was successfully fabricated via a facile aqueous strategy. The obtained ZIF-67 exhibited a high BET specific surface area of 1842.31 m^2^ g^−1^, with a maximum Langmuir adsorption capacity of 156.74 mg g^−1^ toward methyl orange (MO). The adsorption kinetics successfully followed the pseudo-second-order model, and the material maintained 88.9% dye removal efficiency after six ethanol-regeneration cycles. Common inorganic anions (HCO_3_^−^ and SO_4_^2−^) presented a distinct inhibitory effect on MO adsorption. Static electrostatic potential calculations and hydrated molecular dynamics simulations were further adopted to reveal the interfacial adsorption behavior on typical ZIF-67 (100) and (110) facets. Based on equilibrated trajectories of 40–50 ps, both facet systems exhibited stable temperature and potential-energy fluctuations. The dominant Co–O(MO) radial distribution distances for (100) and (110) facets were determined to be 5.825 Å and 5.775 Å, respectively, both far beyond conventional short-range Co–O coordination lengths. The stronger radial ordering of the (110) facet suggests facet-sensitive interfacial organization, rather than direct Co–O chemical bonding during MO adsorption.

## 1. Introduction

With the rapid development of various light industries such as textile, leather, papermaking, printing, and cosmetics industries, water resources have faced increasingly severe challenges [1,2]. Among these, the discharge of dyeing wastewater now constitutes a principal cause of water contamination [3,4,5]. Most dyes in dyeing wastewater are azo-based organic dyes, featuring high chemical stability, strong resistance to photodegradation and oxidative degradation, high concentration, dark chroma as well as carcinogenic, teratogenic and mutagenic risks [6,7,8,9,10]. Often, such dyeing wastewater enters rivers, lakes, and other aquatic environments untreated, poisoning aquatic organisms and even seeping into groundwater, thereby posing a direct threat to human health [11,12,13]. Therefore, the treatment of dyeing wastewater is an urgent issue that needs to be addressed [14,15].

Currently, common methods for removing organic dyes include coagulation–precipitation, adsorption, membrane separation, chemical oxidation, ion exchange, and anaerobic–aerobic microbial degradation [16,17,18]. Among these, the adsorption method is widely used for treating pollutants due to its simplicity of operation, no harmful byproduct generation, good economy, and environmental friendliness [19]. It is particularly effective for adsorbing highly water-soluble and biodegradation-resistant pollutants [20]. Thus, it is crucial to fabricate adsorbents for dye removal from dyeing wastewater.

Metal–organic frameworks (MOFs) represent a material category featuring exceptional surface area density, tunable microporous structures, and diverse pore sizes [21,22]. They are commonly used in gas adsorption, separation, storage, and catalysis [23,24]. As a special type of MOFs, zeolitic imidazolate frameworks (ZIFs) possess excellent chemical stability, thermal stability, and strong adsorption capacity, making them widely applicable for removing water pollutants such as pharmaceuticals and heavy metals [25,26,27]. In the past few years, multiple research efforts have reported the utilization of ZIFs for adsorbing small-molecule organic dyes [28,29]; however, research on the dye adsorption mechanism remains limited, and studies on the competitive adsorption of dyes are even scarcer [30,31].

To the best of the knowledge of the authors, research concerning the green and hierarchical synthesis of ZIF-67 and its application for the adsorption of anionic dyes, such as methyl orange, is scarce. Moreover, the molecular-level adsorption mechanism of MO on ZIF-67 has not been fully elucidated. In this study, a rhombic dodecahedral ZIF-67 was synthesized via a facile and green route, and its adsorption performance toward MO was systematically investigated. The adsorption kinetics, isotherm behavior, and recyclability were evaluated in detail. Furthermore, molecular-level insights into the adsorption mechanism were provided by electrostatic potential (ESP) analysis and molecular dynamics (MD) simulations.

In this study, ZIF-67 was synthesized via a green and facile method and systematically characterized by XRD, FT-IR, SEM/EDS, TGA, and zeta potential measurements. MO was selected as a model anionic dye to evaluate the adsorption performance under various operational conditions, including solution pH, temperature, initial dye concentration, adsorbent dosage, contact time, and the presence of competing ions. The adsorption behavior was further analyzed using kinetic and isotherm models to elucidate the adsorption process. In addition, the adsorption mechanism and selectivity toward MO were thoroughly investigated. The reusability of the adsorbent was also assessed through multiple adsorption–desorption cycles. Furthermore, molecular-level insights into the adsorption mechanism were provided via ESP analysis and MD simulations, enabling a deeper understanding of the interaction between MO and ZIF-67.

## 2. Results and Discussion

### 2.1. XRD Characterization of ZIF-67

**Figure 1:**
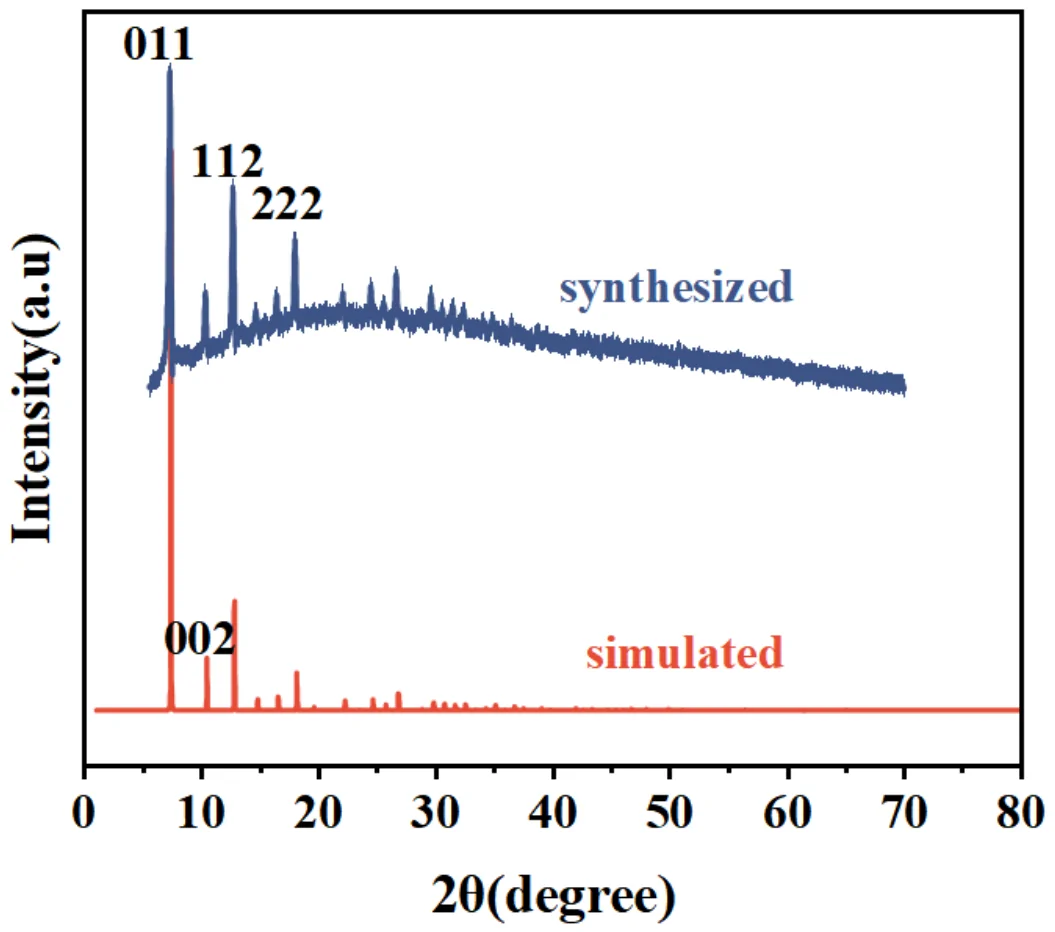
Experimental XRD pattern of synthesized ZIF-67 compared with the simulated pattern calculated from the ZIF-67 CIF.

The crystal structure and phase purity of the synthesized ZIF-67 were examined by X-ray diffraction over 2θ = 4–80°. Figure 1 compares the experimental pattern of the synthesized sample with the simulated pattern calculated from the ZIF-67 crystallographic information file. The experimental peaks at 2θ = 7.37°, 10.43°, 12.79°, 14.77°, 16.53°, 18.12°, 22.24°, 24.62°, 25.73°, 26.80°, 29.80°, 30.74°, and 32.55° were assigned to the (011), (002), (112), (022), (013), (222), (114), (233), (224), (134), (044), (334), and (235) planes, respectively [32]. Their close agreement with the simulated pattern and published data [33] confirms the successful formation and high crystallinity of ZIF-67. The elevated background between 20° and 60° is attributed to diffuse scattering and instrumental background rather than a distinct amorphous phase.

### 2.2. FTIR Characterization of ZIF-67

**Figure 2:**
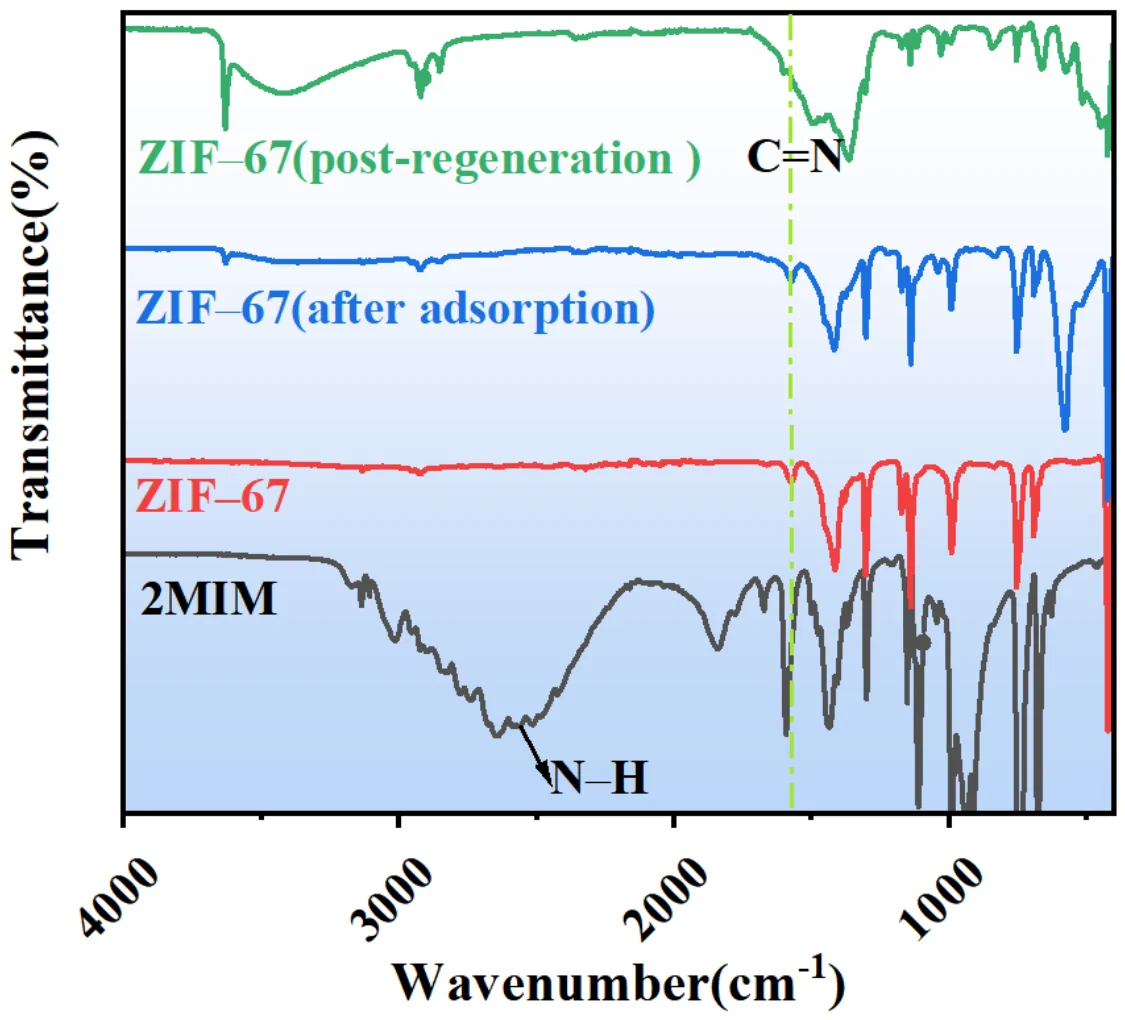
FTIR spectra of 2-methylimidazole (2MIM), as-synthesized ZIF-67, MO-loaded ZIF-67, and regenerated ZIF-67 after ethanol desorption.

Figure 2 compares the FTIR spectra of 2-methylimidazole (2MIM), as-synthesized ZIF-67, MO-loaded ZIF-67, and regenerated ZIF-67 after ethanol desorption. Free 2MIM was included as a reference for assessing the chemical changes accompanying framework formation. It exhibited a broad absorption envelope in the approximately 2500–3500 cm^−1^ region, attributable mainly to hydrogen-bonded N–H stretching, with possible contributions from adsorbed moisture. As shown in Table 1, the relatively sharper band at 3141 cm^−1^ is assigned to aromatic C–H stretching. The marked attenuation of the broad N–H-associated feature in ZIF-67, together with the appearance of the Co–N stretching band near 420 cm^−1^, is consistent with deprotonation of 2MIM and coordination of Co^2+^ with imidazolate nitrogen atoms. The band at 1583 cm^−1^ is assigned predominantly to C=N stretching, whereas those at 1422 and 1305 cm^−1^ arise from imidazolate-ring stretching and deformation. The bands at 1135 and 987 cm^−1^ are associated with C–N stretching coupled with in-plane ring vibrations, while those at 754 and 690 cm^−1^ correspond primarily to out-of-plane ring deformation. The band at 2928 cm^−1^ is assigned to aliphatic C–H stretching. These assignments agree with reported FTIR spectra of ZIF-67, particularly the characteristic C=N and Co–N bands near 1580 and 420 cm^−1^, respectively [34].

After MO adsorption, the principal ZIF-67 bands—including the C=N band near 1580 cm^−1^, imidazolate-ring modes near 1420 and 1305 cm^−1^, C–N/ring modes near 1140 and 990 cm^−1^, and Co–N band near 420 cm^−1^—remained detectable without pronounced shifts or disappearance. This observation indicates that the majority of the local bonding environment within ZIF-67 was retained during adsorption. An enhanced feature was observed at approximately 576 cm^−1^ in the MO-loaded sample. Because this region may contain overlapping contributions from the framework, adsorbed dye, and interfacial vibrations, the feature is conservatively interpreted as an MO-associated change in the interfacial vibrational environment rather than direct evidence of Co–O coordination [35]. We cannot fully rule out minor Co-O interactions at the interface; nevertheless, this FTIR band alone cannot provide definitive proof for such coordination. Relative band intensities were not interpreted quantitatively, given the qualitative nature of these spectra and potential confounding effects from sample packing, light scattering, and dye loading. After ethanol regeneration, the principal C=N, C–N/ring, and Co–N bands remained detectable, indicating preservation of key local bonding features following adsorption–desorption cycling. Taken together with independent adsorption measurements that describe the uptake and recyclability performance, these FTIR results support MO adsorption while demonstrating that the local bonding environment of ZIF-67 is largely preserved.

### 2.3. SEM/EDS Characterization of ZIF-67

**Figure 3:**
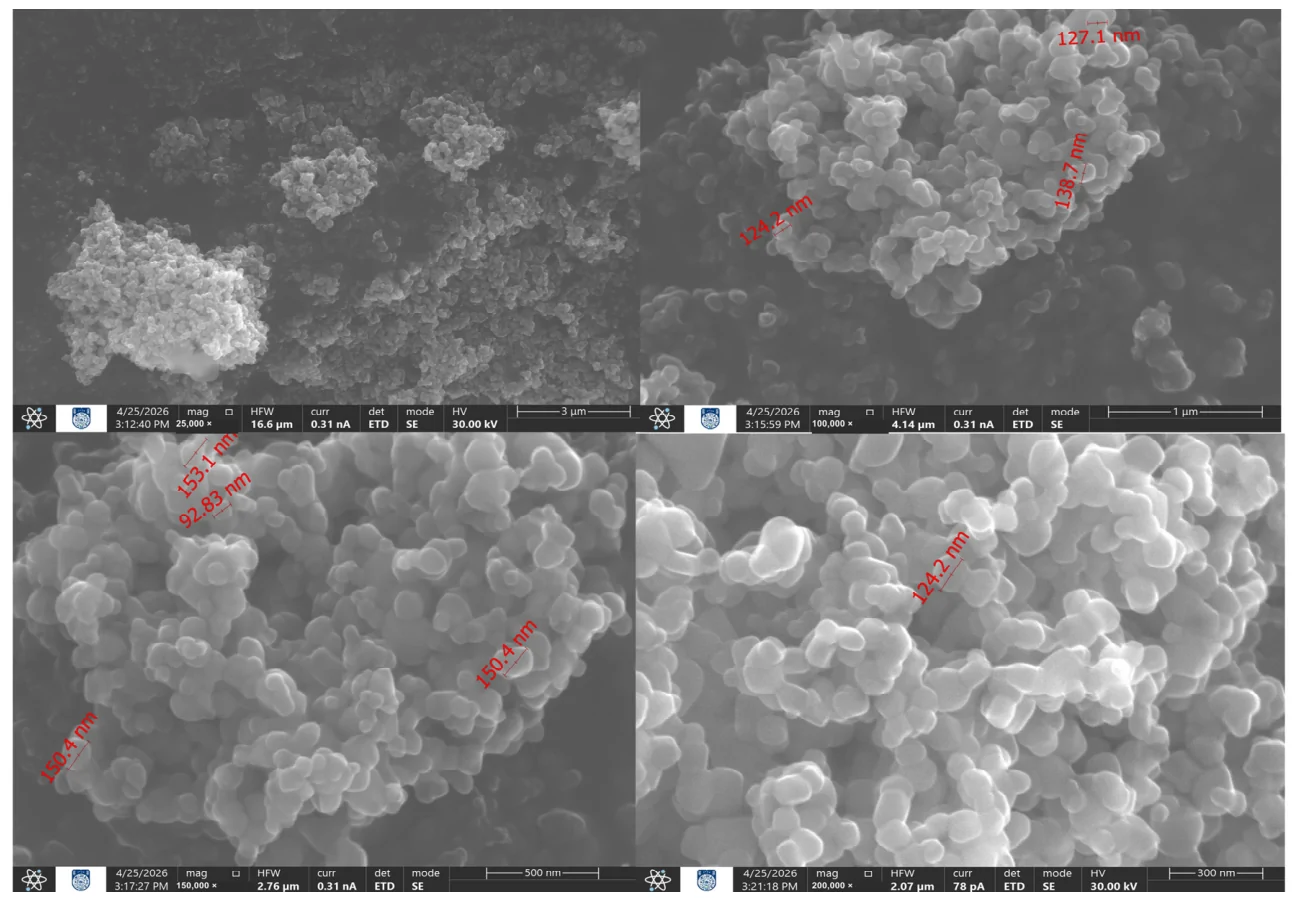
SEM images of ZIF-67.

The ZIF-67 SEM images are displayed in Figure 3. The samples were dispersed in ethanol prior to the analysis. Then the dispersion was dropped on the carbon tape attached to the aluminum stub. For the enhancement of conductivity and to avoid charging effects, the samples were sputter-coated with a thin layer of carbon prior to the analysis using the SEM. The high-magnification SEM image (×200,000) in Figure 3 shows that the ZIF-67 exhibits good dispersibility, small and uniform particle size (approximately 100–200 nm), and regular morphology, which is consistent with the standard rhombic dodecahedral structure reported in the literature [36].

**Figure 4:**
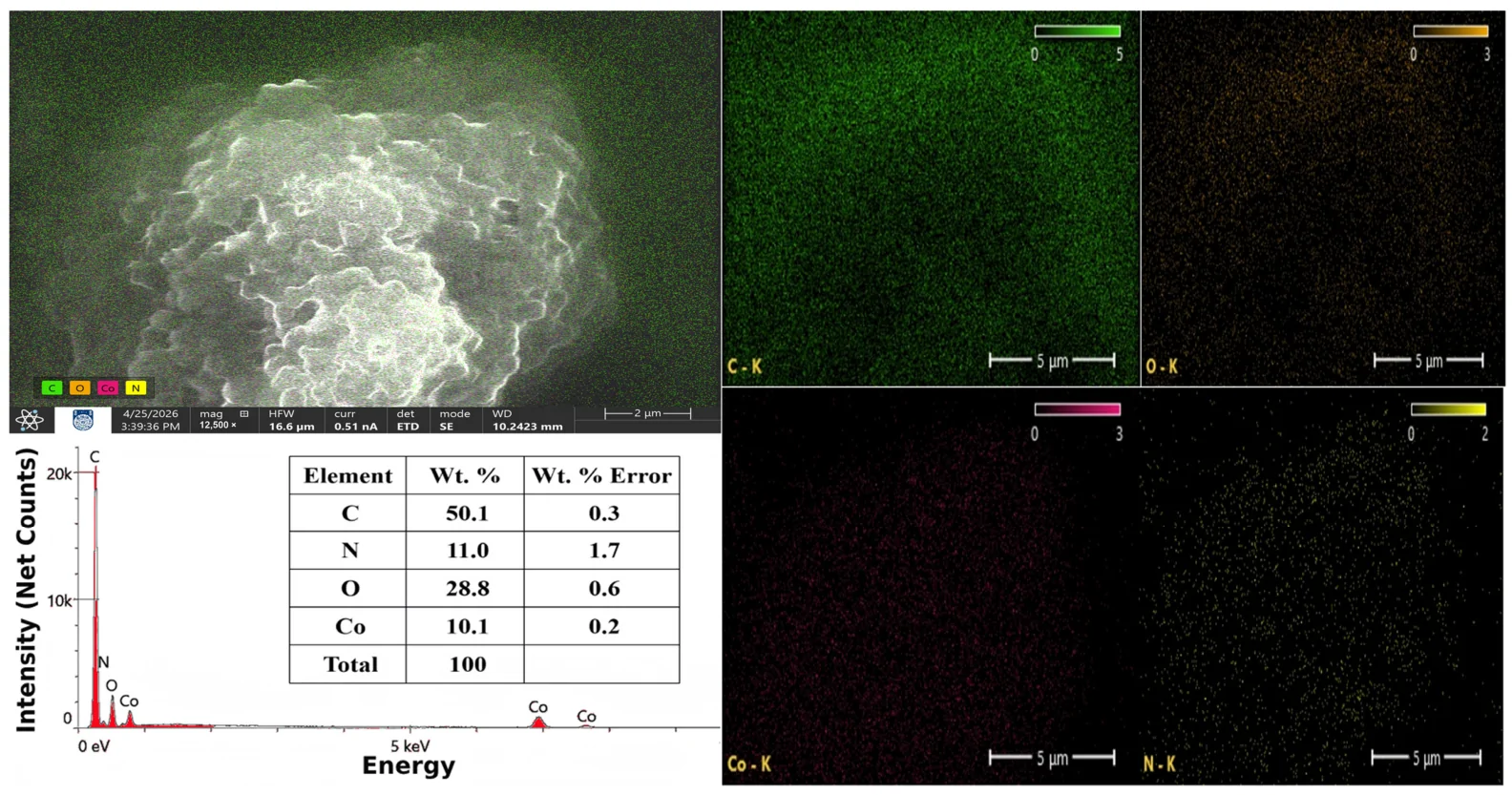
EDS and Mapping analyses of the ZIF-67.

Figure 4 presents electron scattering spectroscopy, mapping images, and elemental quantitative data for a more thorough analysis of ZIF-67 particles. According to EDX elemental analysis, carbon, nitrogen, oxygen, and cobalt are present in ZIF-67. Carbon (50.1 wt.%) and nitrogen (11.0 wt.%) are the major elements present due to the 2-methylimidazole molecule, whereas oxygen (28.8 wt.%) and cobalt (10.1 wt.%) account for surface-adsorbed oxygen-containing molecules and cobalt ions, respectively. The findings show that these components are uniform over the surface. EDS analysis shows that cobalt is the main metallic element, while the carbon content is relatively high. Additionally, the elements exhibit certain spatial distribution characteristics in the sample.

### 2.4. TGA Characterization of ZIF-67

**Figure 5:**
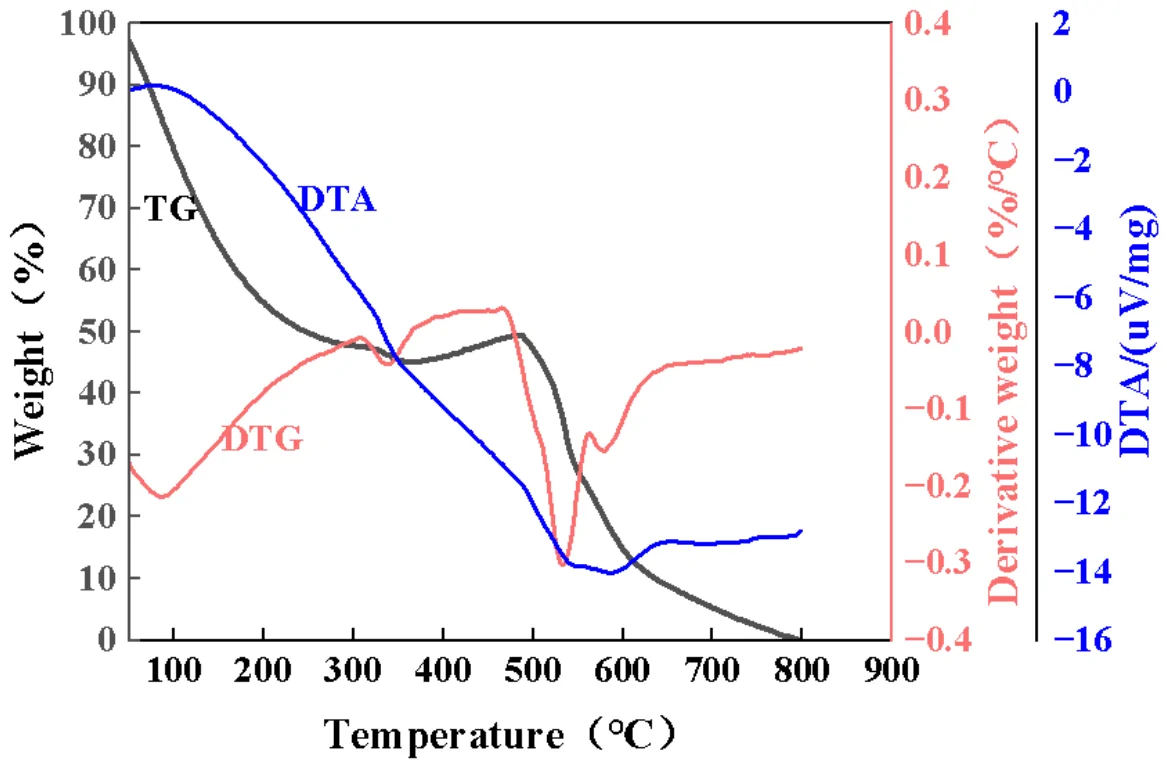
TGA, DTA and DTG curve of ZIF-67.

The thermal stability of as-prepared ZIF-67 was examined by synchronous TG-DTG-DTA under nitrogen atmosphere. Different temperature transitions that were indicative of their structural stability and disintegration mechanisms were found by examining the TGA data shown in Figure 5. The DTG and DTA curves in the figure were obtained directly from the raw data, whereas the TG data were normalized in relation to the initial weight of the ZIF-67 powder. From the result depicted in the graph, it is clear that the thermal decomposition of ZIF-67 consists of three major steps. The first step takes place between room temperature and 350 °C which corresponds to desorption of absorbed water, residual solvent, and guest molecule in the pores of ZIF-67 with strong endothermic peak at 331.5 °C. The second step ranges from 350 to 550 °C in which the ZIF-67 framework starts to collapse and the 2-methylimidazole starts to thermally decompose and carbonize with two endothermic peaks at 416.6 °C and 498.5 °C, respectively, and DTG exhibits the peak weight loss [37]. The third step occurs above 550 °C in which the remaining carbonaceous compounds and inorganic phase [7] containing cobalt start to evolve with weak exothermic peaks at 607.6 °C and 794.6 °C, respectively. Finally, the weight percent of the residue at the temperature of 800 °C was found to be 45.18%. The results demonstrate that ZIF-67 exhibits strong thermal stability.

### 2.5. BET Characterization of ZIF-67

**Figure 6:**
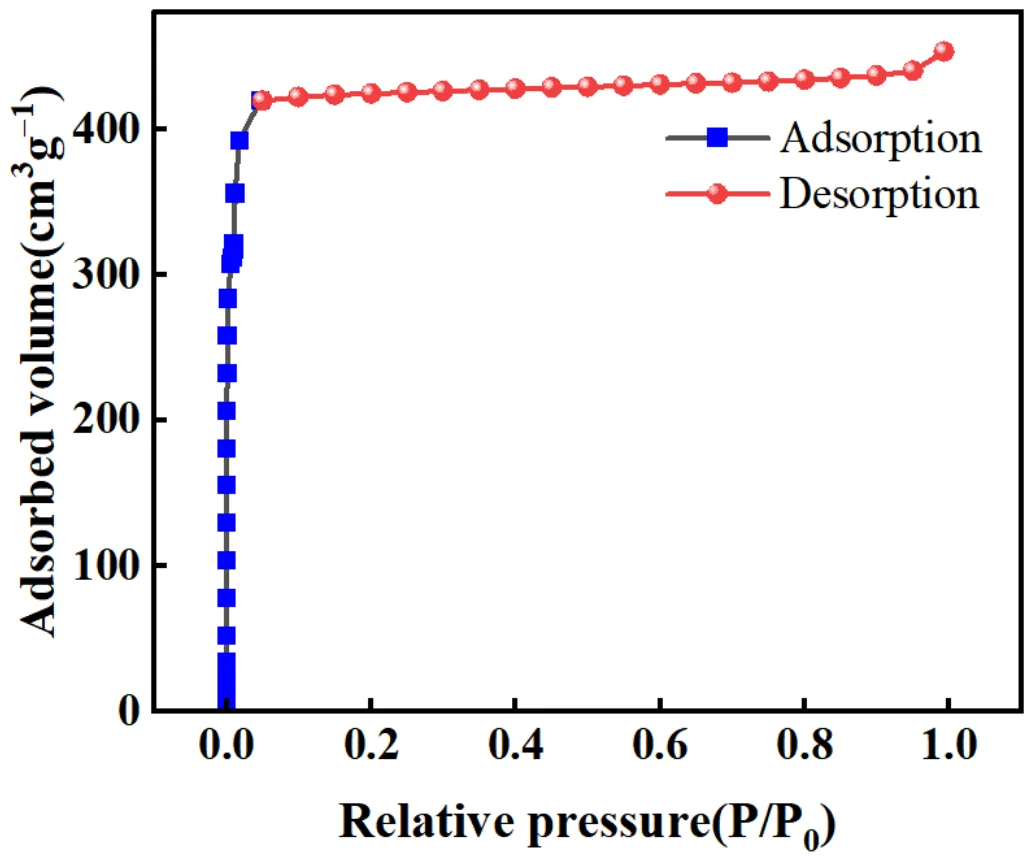
BET curve of ZIF-67.

Type I adsorption/desorption isotherm features are observed in the N_2_ adsorption/desorption isotherm of ZIF-67 (Figure 6). The abrupt rise in the amount of adsorbed gas at lower relative pressures (P/P0 < 0.05) proves that there are numerous micropores, which is a feature of the ZIF-67-based samples [38]. The adsorption curve quickly achieves saturation beyond (P/P0 > 0.1) without any indication of a hysteresis loop, implying that the material is devoid of mesopores and macropores. The lack of a hysteresis loop further substantiates the highly reversible nature of the adsorption process in the microporous network [39]. The BET surface area of ZIF-67 is 1842.31 m^2^/g. ZIF-67’s specific surface area and pore structure significantly influence its adsorption ability [40]. In addition to micro and mesoporous structures, the interactions between the dye and ZIF-67 include π–π stacking interactions and electrostatic interactions.

### 2.6. Effect of Experimental Parameters on Methyl Orange Adsorption by ZIF-67

#### 2.6.1. Effect of Adsorbent Dosage and Initial Concentration

**Figure 7:**
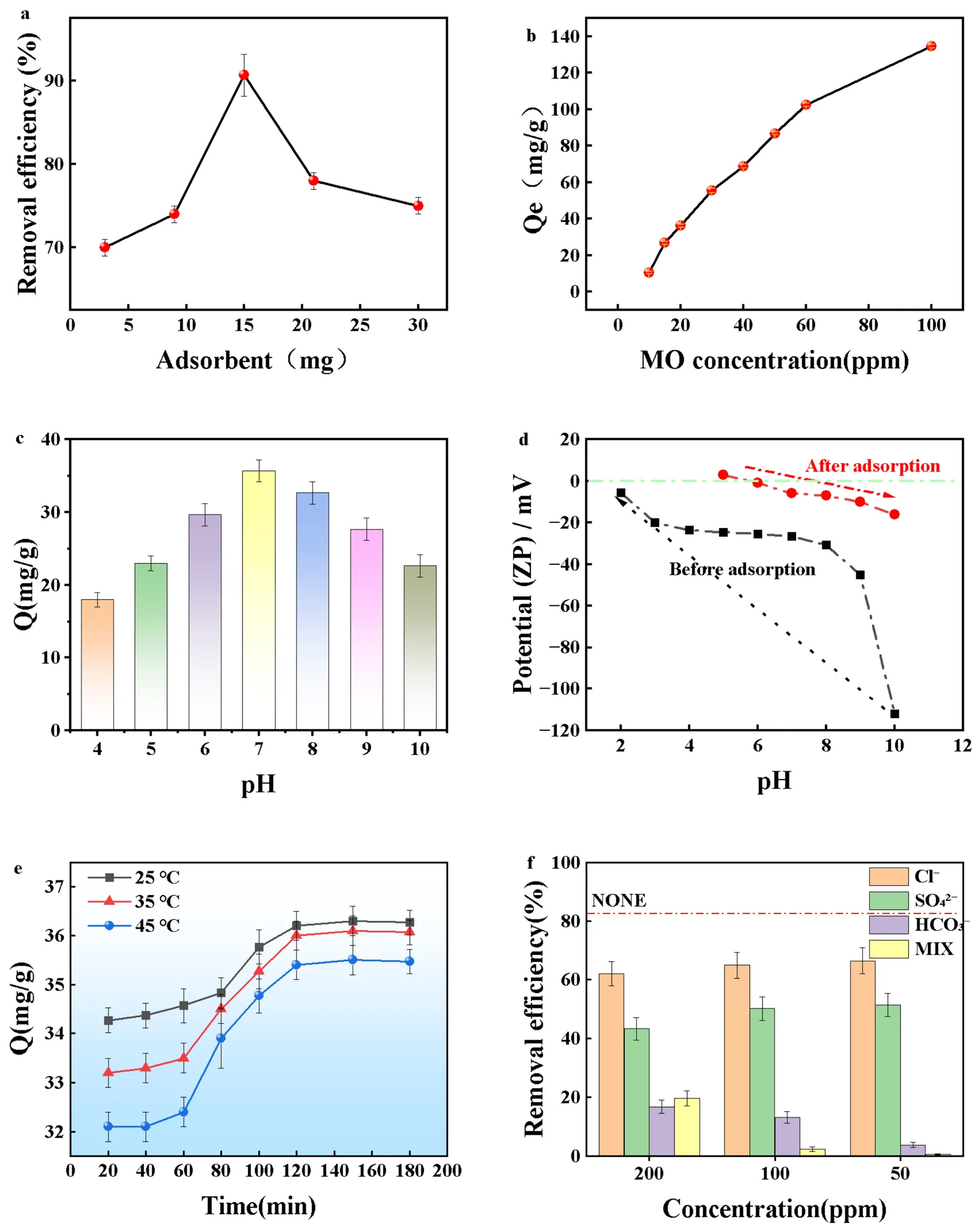
Effects of (**a**) ZIF-67 dosage on MO removal at an initial MO concentration of 20 mg L^−1^; (**b**) initial MO concentration on adsorption capacity; (**c**) solution pH on adsorption capacity at 20 mg L^−1^ MO and 15 mg ZIF-67; (**d**) zeta potential before and after MO adsorption; (**e**) contact time and temperature on adsorption capacity; and (**f**) competing-ion concentration (20–200 mg L^−1^) on MO removal at a fixed initial MO concentration of 20 mg L^−1^. Data are means of at least three independent experiments; error bars show standard deviations.

Adsorbent dosage was evaluated at an initial MO concentration of 20 mg L^−1^ while pH, contact time, and solution volume were held constant. As shown in Figure 7a, MO removal increased with ZIF-67 dosage and reached 91% at 15 mg because the number of accessible adsorption sites increased [41]. A slight decline at higher dosages may reflect particle aggregation and overlap of available sites. Accordingly, 15 mg was selected as the optimum dosage.

The adsorption capacity of the adsorbent is strongly influenced by the starting concentration of the MO dye. As depicted in Figure 7b, at an adsorbent dosage of 15 mg, the adsorption capacity increases with increasing initial methyl orange concentration. This is because a higher initial concentration increases the concentration gradient between the adsorbent surface and the solution bulk, providing a stronger driving force for adsorption and allowing more adsorption sites on the ZIF-67 surface to bind with methyl orange [42]. Once the initial concentration exceeds 20 mg/L, the adsorption capacity increases gradually, as most adsorption sites on the adsorbent surface are occupied by methyl orange, and the available adsorption sites decrease, leading to a tendency toward saturation [43]. In conclusion, the increased driving force at higher dye concentrations benefits the adsorption capacity, which shows a favorable trend.

#### 2.6.2. Effect of pH

Solution pH can affect adsorption by changing the protonation state and interfacial charge environment of the adsorbent, the speciation of the adsorbate, and the accessibility of surface binding sites, as well as framework stability. For MO, however, the sulfonate group remains predominantly dissociated over the investigated pH range. Therefore, the pH dependence of adsorption should not be attributed to protonation and deprotonation of the sulfonate group. Instead, it may reflect changes in the protonation state of ZIF-67 surface sites, pH-dependent electronic structure of the MO azo chromophore, competition from solution ions, and pH-dependent framework stability.

The effect of initial solution pH (x-axis in Figure 7c corresponds to initial pH before ZIF-67 addition) was investigated using an MO concentration of 20 mg L^−1^ and 15 mg of ZIF-67, while the other experimental conditions were held constant. As shown in Figure 7c, the adsorption capacity increased from pH 4 to 7 and subsequently decreased between pH 7 and 12. MO remains anionic because of its dissociated sulfonate group throughout most of this range; protonation of the azo-containing chromophore mainly occurs near strongly acidic conditions close to pH 4 and can alter its electronic structure and interfacial interactions [44]. The increase in adsorption up to pH 7 may therefore reflect a favorable balance among MO speciation, the protonation state of accessible ZIF-67 surface sites, and framework stability. At higher pH, progressive deprotonation of surface sites, together with increasing competition from OH^−^ and changes in the interfacial electrostatic environment, as well as potential partial framework degradation, may reduce MO uptake. Under the tested conditions, pH 7 produced the highest adsorption capacity and was therefore selected for the subsequent experiments.

Zeta-potential measurements were used to examine changes in the interfacial charge environment after adsorption. As shown in Figure 7d, the zeta potential of ZIF-67 shifted toward less negative values after exposure to MO. This shift indicates that adsorption altered the composition and charge distribution of the particle–solution interface [45,46]. Notably, given that MO carries negatively charged sulfonate groups, the observed shift cannot be interpreted as simple charge neutralization, nor can zeta-potential data alone constitute direct proof of MO adsorption. The change may also reflect dye molecular orientation, counterion association, displacement of surface-bound ions, or modification of the slipping plane, or even interfacial changes originating from material–solvent interactions. Accordingly, the zeta-potential result is treated only as supporting evidence for interfacial modification and is interpreted together with adsorption measurements and FTIR results.

#### 2.6.3. Effect of Contact Time and Temperature

As shown in Figure 7e, the adsorption capacity curves over a temperature range of 25–45 °C exhibit similar trends. The adsorption capacity of ZIF-67 for methyl orange increases rapidly with increasing contact time and stabilizes after 120 min, indicating that higher temperatures are unfavorable for adsorption; hence it is an exothermic process [10].

#### 2.6.4. Effect of Competing Ions

In the competing-ion experiments (Figure 7f), the values 20, 100, and 200 mg L^−1^ refer to the concentrations of the added competing ions, whereas the initial MO concentration was fixed at 20 mg L^−1^. In the absence of added salt, the MO removal efficiency was 81%. In the presence of NaCl, the removal efficiency ranged from 62% to 67%, whereas Na_2_SO_4_ reduced it to 43–56%. NaHCO_3_ caused more pronounced inhibition, and the mixed-salt system produced the lowest removal efficiency.

Under the tested mass-concentration conditions, the apparent inhibition followed the order mixed salts > NaHCO_3_ > Na_2_SO_4_ > NaCl. The relatively moderate effect of NaCl is consistent with limited perturbation of the MO/ZIF-67 interface under the present conditions [47]. The stronger inhibition caused by Na_2_SO_4_ may arise from competition between SO_4_^2−^ and anionic MO for accessible interfacial regions, together with enhanced electrostatic screening originating from the divalent anion [48]. In addition to anion competition, NaHCO_3_ may alter the solution pH through buffering and thereby modify the protonation state and interfacial environment of ZIF-67 surface sites; potential local framework perturbation induced by bicarbonate cannot be ruled out. Its effect should not be attributed to conversion of MO from a neutral species to an anion because the sulfonate group of MO remains predominantly dissociated over the relevant pH range. The lowest removal observed in the mixed-salt system is consistent with the simultaneous influence of multiple competing ions and associated changes in solution chemistry. Because different salts were compared on an equal-mass-concentration basis rather than equal anion molar concentrations or ionic strengths, this sequence represents an empirical inhibition order under the tested conditions rather than an intrinsic affinity ranking of Cl^−^, SO_4_^2−^, and HCO_3_^−^ for ZIF-67.

### 2.7. Sorption Kinetics Studies

The adsorption kinetics of MO onto ZIF-67 were evaluated using the pseudo-first-order (PFO) and pseudo-second-order (PSO) models. The PFO model was originally developed to describe the time-dependent approach of adsorption capacity toward equilibrium, whereas the PSO model expresses the adsorption rate as a function of the square of the difference between the equilibrium and time-dependent adsorption capacities [49,50]. Ho and McKay systematically compared these kinetic models and demonstrated the importance of evaluating multiple rate equations rather than assigning a kinetic mechanism on the basis of a single model [49]. The PFO and PSO models have also been used in recent adsorption studies to compare the calculated equilibrium adsorption capacity with the experimentally measured value [50].

(1) $$\ln\left(q_{e}-q_{t}\right)=\ln q_{e}-k_{1}t$$

(2) $$\frac{t}{q_{t}}=\frac{1}{k_{2}q_{e}^{2}}+\frac{t}{q_{e}}$$

where:

*k*_1_ = pseudo-first-order rate constant (min^−1^);

*k*_2_ = pseudo-second-order rate constant (g mg^−1^ min^−1^);

*t* = contact time (min).

The PFO parameters *k*_1_ and *q_e_* were obtained by fitting ln(*q_e_* − *q_t_*) versus *t*, whereas the PSO parameters *k*_2_ and *q_e_* were obtained by fitting *t*/*q_t_* versus *t*.

**Figure 8:**
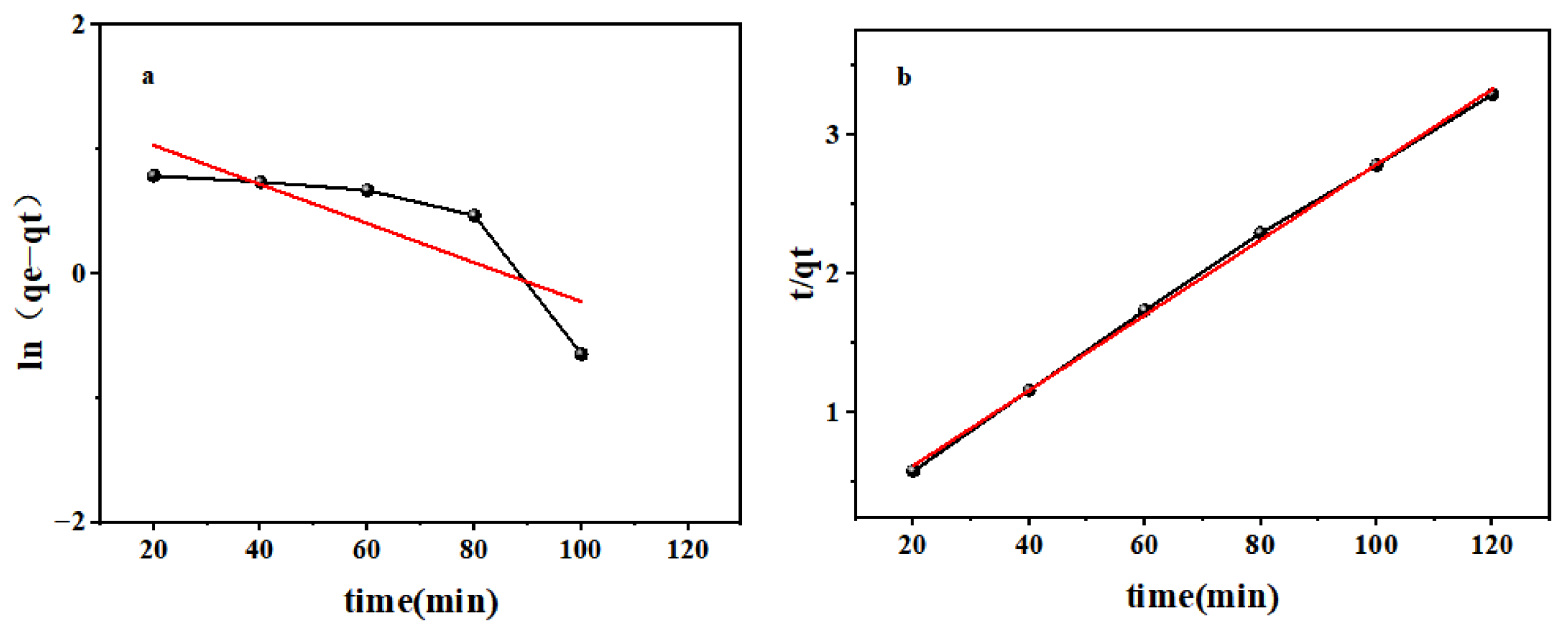
Pseudo-first-order (**a**) and pseudo-second-order (**b**) kinetic fitting curves for methyl orange adsorption by ZIF-67. Black symbols represent experimental data; red solid lines are the corresponding model fitting curves. Data represent mean values from triplicate experiments (*n* ≥ 3) with standard deviations shown as error bars.

The adsorption process of methyl orange was fitted using the two kinetic models, and the fitting curves are shown in Figure 8.

As shown in Table 2 and Table 3, the adsorption kinetics were evaluated using the pseudo-first-order (PFO) and pseudo-second-order (PSO) models, respectively. At 25 °C, the PSO model exhibited a higher coefficient of determination than the PFO model. In addition, the equilibrium adsorption capacity calculated using the PSO model was closer to the experimentally measured value. These results indicate that the PSO equation provides a better empirical description of the MO adsorption kinetics under the investigated conditions. However, agreement with the PSO model alone does not establish chemisorption as the exclusive adsorption mechanism because kinetic model fitting cannot independently distinguish among surface reaction, mass transfer, and diffusion effects. Therefore, the kinetic results were interpreted together with the Weber–Morris intraparticle-diffusion analysis and the experimental and molecular characterization results.

The Weber–Morris intraparticle-diffusion model was further applied to examine the contribution of mass transfer to the adsorption rate. If the plot of q_t_ versus t^1/2^ is linear and passes through the origin, intraparticle diffusion may be considered the principal rate-controlling step. In the present study, the plot contained two linear regions and neither regression line passed through the origin. The first region can be associated with relatively rapid external-surface adsorption and boundary-layer transport, whereas the second region represents slower diffusion toward less accessible adsorption sites. The nonzero intercepts indicate that intraparticle diffusion was involved but was not the sole rate-controlling process. The mathematical expression for this relationship is:

(3) $$q_{t=k_{\mathrm{id}}}.t^{1/2}+C$$

where:

q_t_ = amount of MO adsorbed at time t (mg g^−1^);

t^1/2^ = square root of contact time (min^1/2^);

k_id_ = intraparticle-diffusion rate constant obtained from the slope (mg g^−1^ min^−1/2^);

C = intercept associated with the boundary-layer contribution (mg g^−1^).

Based on this approach, where there is linearity and the graph passes through the origin (where C = 0), only intraparticle diffusion governs the adsorption rate. On the other hand, the presence of a multilinearity graph indicates that there are two or more governing processes taking part in the adsorption rate process, such as external mass transport and intraparticle diffusion. Furthermore, if there is linearity but it does not pass through the origin (where C > 0), then boundary layer diffusion also affects the adsorption process rate.

**Figure 9:**
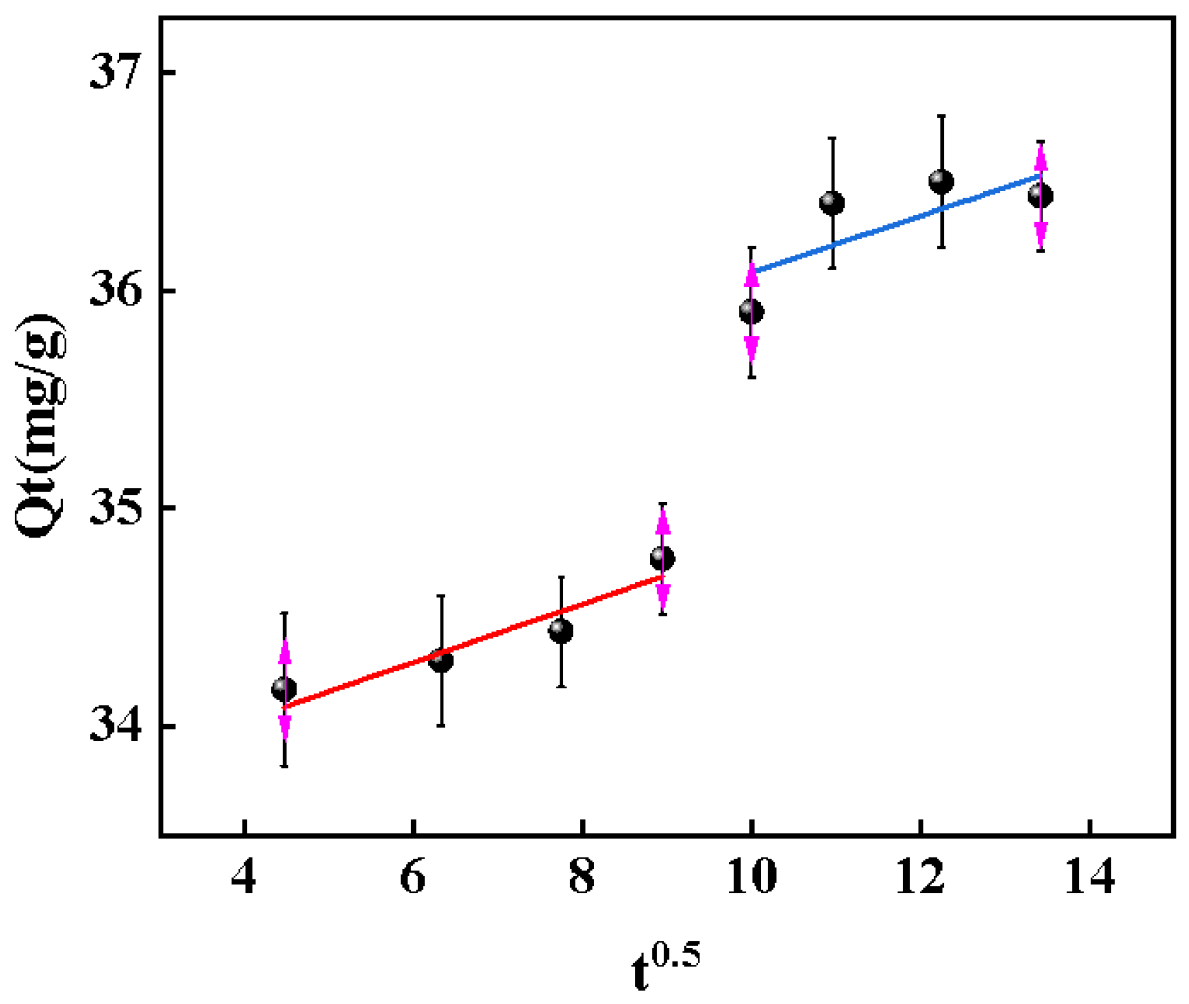
Weber–Morris intra-particle diffusion model for methyl orange adsorption by ZIF-67. Black spheres denote experimental data; the red and blue solid lines represent linear fitting results for two separate diffusion stages. The magenta double-headed arrows indicate the boundary between different diffusion stages. Data represent mean values from triplicate experiments (*n* ≥ 3) with standard deviations shown as error bars.

The Weber–Morris intraparticle diffusion model has been used to study the adsorption kinetics of MO adsorption onto ZIF-67, and the results are shown in Figure 9. It is observed from the figure that the plot of q_t_ versus t^1/2^ is a straight line, indicating that the adsorption kinetic process is not controlled by a single step [51]. The first linear part of the plot corresponds to the external diffusion step, in which MO adsorption occurs rapidly, and the adsorption rate is controlled by boundary layer diffusion. The second linear part of the plot corresponds to the intraparticle diffusion step, in which MO adsorption occurs gradually, and the adsorption rate is reduced due to the diffusion resistance. It is observed from the figure that neither of the two linear segments passes through the origin, indicating that the adsorption kinetic process is not controlled by a single step, and the intraparticle diffusion is not the only rate-controlling step in the adsorption kinetic process.

### 2.8. Adsorption Isotherms

The equilibrium link between the amount of MO adsorbed by the adsorbent and its concentration in the solution at a constant temperature is described using adsorption isotherm models. Adsorption isotherm models were used to fit the experimental data and investigate the adsorption behavior of methyl orange on the ZIF-67 surface. The Langmuir and the Freundlich models were chosen because of their general use and their ability to represent idealized homogeneous and heterogeneous adsorption systems. The Langmuir and the Freundlich models are represented by the following equations.

(4) $$\frac{c_{e}}{q_{e}}=\frac{c_{e}}{q_{m}}+\frac{1}{q_{m}K_{L}}$$

(5) $$\ln q_{e}=\ln K_{F}+\frac{1}{n}\ln c_{e}$$

where

*q_m_* = Langmuir fitted maximum adsorption capacity (mg g^−1^);

*K_L_* = Langmuir equilibrium constant (L mg^−1^);

*K_F_* = Freundlich adsorption-capacity constant (with units determined by n);

*n* = Freundlich adsorption-intensity exponent.

The Langmuir parameters *q_m_* and *K_L_* were obtained by fitting *c_e_*/*q_e_* versus *c_e_*, whereas *K_F_* and *n* were obtained by fitting ln*q_e_* versus ln*c_e_*.

**Figure 10:**
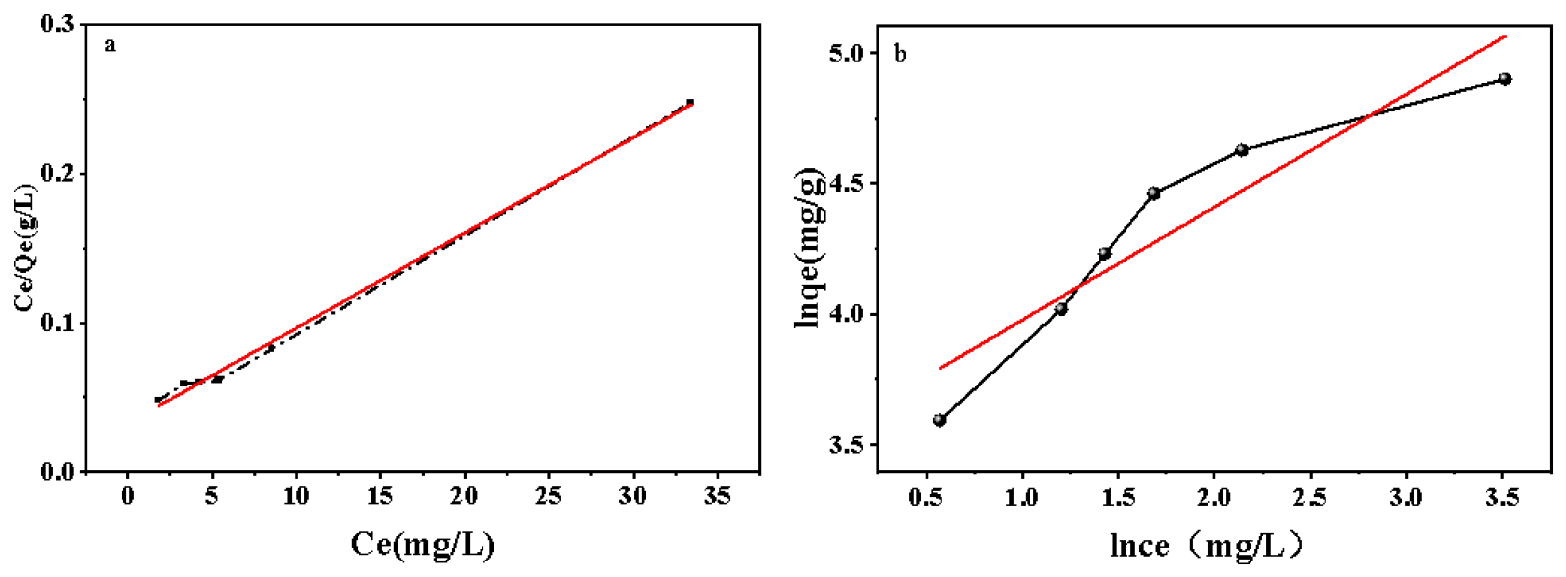
Langmuir (**a**) and Freundlich (**b**) isotherms for methyl orange adsorption by ZIF-67. Black symbols represent experimental data; black dashed line (**a**) and black solid line (**b**) connect experimental points; red solid lines are the corresponding model fitting curves. Data represent mean values from triplicate experiments (*n* ≥ 3) with standard deviations shown as error bars.

The experimental data of methyl orange adsorption by ZIF-67 were fitted using the Langmuir and the Freundlich models, and the fitting curves are shown in Figure 10.

As summarized in Table 4, the Langmuir model provided a consistently better fit than the Freundlich model over the investigated temperature range of 25–45 °C. The corresponding R^2^ values for the Langmuir model were 0.996, 0.969, and 0.970 at 25, 35, and 45 °C, respectively, compared with 0.846, 0.958, and 0.9534 for the Freundlich model. These results suggest that monolayer adsorption was predominant under the investigated conditions.At 25 °C, the maximum adsorption capacity predicted by the Langmuir model was 156.74 mg g^−1^, whereas the highest experimentally measured capacity was 137.68 mg g^−1^, corresponding to 87.8% of the predicted value. The difference is reasonable because the experimental concentration range may not have saturated every accessible site and because particle heterogeneity, mass-transfer limitations, and experimental uncertainty are not represented by the ideal Langmuir assumptions. The reasonable consistency between the two sets of fitting results supports the applicability of the Langmuir model to describe the adsorption process.

To further evaluate the adsorption performance of ZIF-67, its maximum adsorption capacity was compared with that of other reported adsorbents for methyl orange removal. As summarized in Table 5, the adsorption capacity of ZIF-67 (156.74 mg/g) is comparable to or higher than many previously reported materials, such as [52,53,54]. This superior performance can be attributed to its high surface area and abundant active sites, which facilitate strong interactions with MO molecules. Although 160-MIL-101 exhibits a higher adsorption capacity than the current material, it was synthesized at 160 °C, and its adsorption process took 24 h [55]. The synthesis of MOR-1-HA still requires purification by HCl acid treatment, reflux heating for 1 h, and the introduction of alginic acid as a flocculating agent [56]. In contrast, our material can be synthesized under room temperature without any organic solvent, offering superior green chemistry metrics. In contrast, the synthesis of MOF-235 suffers from several obvious limitations, including the use of a large amount of toxic DMF solvent, high-pressure autoclave reaction (24 h), high-temperature activation at 150 °C, and centrifugation-based separation [57]. Although MIL-100(Fe) exhibits excellent performance, its synthesis requires the use of highly toxic and corrosive HF, reaction in an autoclave at 160 °C, and relatively complicated product separation steps [58]. On the other hand, the preparation process of UiO-66 derivatives is limited by several disadvantages: high consumption of the toxic solvent DMF, autoclave process under high pressure (12 h), centrifugation for separation, high-temperature dehydration process in vacuum conditions (60 °C, 8 h), and repeated washing with DMF and acetone solutions. Moreover, the preparation of U-B-HCl, a further 12 h is required to treat with HCl [59].

### 2.9. Thermodynamics

The thermodynamic properties of adsorption processes are determined using three parameters: Gibbs free energy (ΔG), entropy (ΔS), and enthalpy (ΔH) [63]. Thermodynamic parameters were calculated to evaluate the spontaneity and nature of the adsorption process [64]. The following equations can be used to determine the Gibbs free energy.

(6) ΔG=ΔH − TΔS

(7) $$\Delta G=-\mathrm{RT}\ln K_{d}$$

The operational distribution coefficient K_d_ at different adsorption temperatures is expressed as follows.

(8) $$K_{d}=\frac{\left(C_{i}-C_{e}\right)}{C_{e}}$$

*C_i_* and *C_e_* represent the solute’s initial and equilibrium concentrations, respectively, in the equation above.

(9) $$\ln K_{d}=\frac{\Delta H}{\mathrm{RT}}-\frac{\Delta S}{R}$$

Accordingly, ΔH and ΔS were obtained from the slope (−ΔH/R) and intercept (ΔS/R), respectively, of the linear regression of lnK_d_ against 1/T. Because K_d_ was defined using an operational concentration ratio rather than a rigorously standardized thermodynamic equilibrium constant, the resulting values are interpreted as apparent thermodynamic parameters.

As shown in Table 6, the apparent thermodynamic parameters of the adsorption of MO onto ZIF-67 yielded negative ΔG° values for adsorption at all studied temperatures: −23.73 kJ/mol, −24.29 kJ/mol, and −24.74 kJ/mol for 298 K, 308 K, and 318 K, respectively. Within the adopted definition of K_d_ these negative values indicate thermodynamically favorable MO uptake under the investigated conditions.

The negative ΔH value (−8.67 kJ mol^−1^) is consistent with an exothermic adsorption process, in agreement with the experimentally observed decrease in equilibrium adsorption capacity with increasing temperature. Although ΔG became numerically more negative as temperature increased, this trend should not be interpreted as evidence that heating enhanced adsorption. The negative ΔH value and the observed decrease in adsorption capacity therefore support more favorable MO uptake at lower temperatures. The relatively small magnitude of the fitted ΔH value is compatible with weak interfacial interactions, but it cannot by itself distinguish physical adsorption from chemisorption or identify a unique adsorption mechanism.

Moreover, the fact that ΔS° is positive +50.5 J/(mol·K) indicates a positive apparent entropy contribution under the adopted model. This may reflect changes in solvent organization, counterion distribution, and interfacial degrees of freedom during adsorption. However, the present thermodynamic data do not independently demonstrate desorption of water molecules or quantify the individual entropy contributions.

Therefore, the fitted parameters are consistent with thermodynamically favorable and exothermic MO adsorption under the investigated conditions. Together with the experimentally observed decrease in adsorption capacity with increasing temperature, the results indicate that lower temperatures favor MO uptake. Because the calculation employed an operational distribution coefficient, these parameters should be regarded as apparent thermodynamic descriptors rather than definitive mechanistic evidence.

### 2.10. Desorption–Adsorption Experiments

Stability and reusability are important considerations when using adsorbents on an industrial scale [65]. In addition to reducing costs, the production of high-performance reusable adsorbents helps minimize the requirements of costly regeneration processes in removing contaminated water [66]. The adsorbent’s strength and removal capabilities are improved by ZIF-67’s strong physical and chemical stability as well as its big surface area. This makes it possible to use the composite in subsequent adsorption cycles.

**Figure 11:**
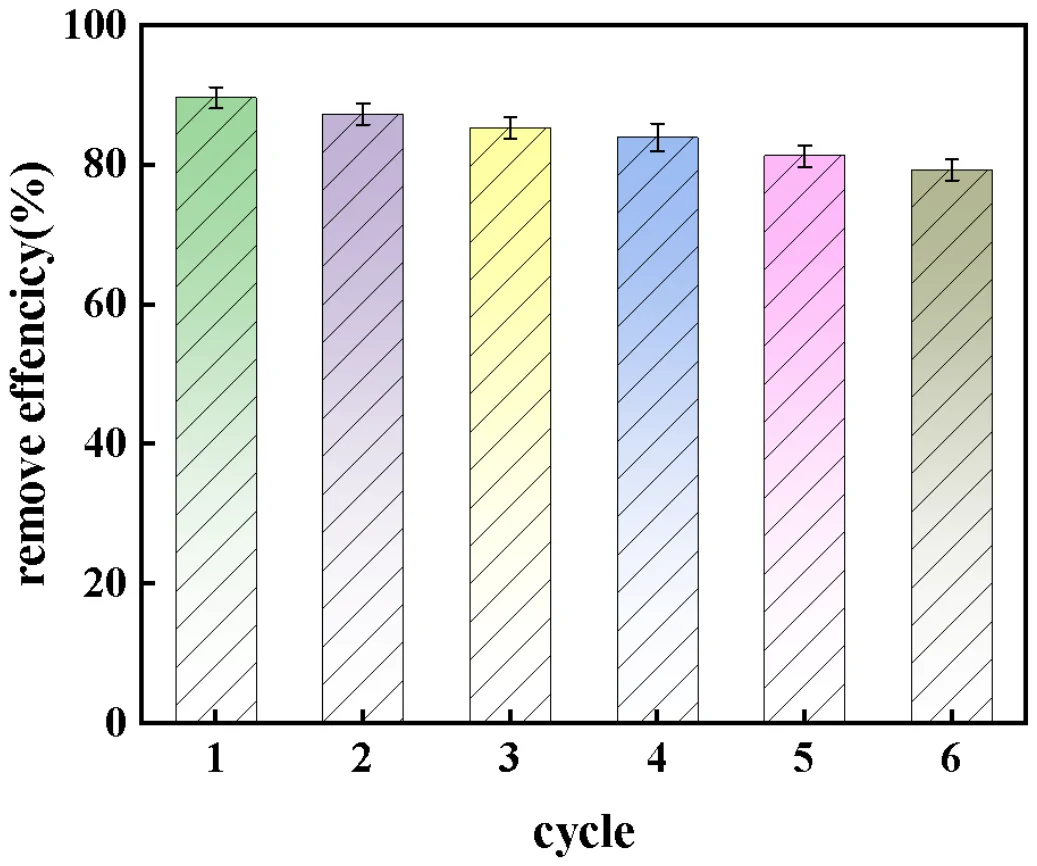
Methyl orange removal rate over different adsorption–desorption cycles. Data represent mean values from triplicate experiments (*n* ≥ 3) with standard deviations shown as error bars.

In this study, ZIF-67 was subjected to six adsorption–desorption cycles using ethanol as the regeneration solution, as shown in Figure 11. As observed, the methyl orange removal rate reaches 98.8% after the first cycle; with increasing cycle times, the removal rate decreases slightly but remains at 88.9% after the sixth cycle. These results illustrated that ZIF-67 can be regenerated by this simple method and maintains a good adsorption capacity after multiple cycles, making it an efficient and economical adsorbent.

Following desorption, the spent regeneration solution consisted primarily of ethanol containing desorbed MO. In practical applications, this solution should not be discharged directly. Instead, ethanol can be recovered by conventional distillation and reused in subsequent regeneration cycles, thereby reducing solvent consumption. After ethanol recovery, the concentrated MO-containing residue can be further treated using established wastewater treatment technologies, such as advanced oxidation processes, photocatalytic degradation, or high-temperature incineration. Although the present study did not experimentally investigate solvent recycling, these strategies provide feasible approaches for minimizing secondary waste generation and improving the environmental sustainability of the adsorption–desorption process.

### 2.11. Evidence-Bounded Adsorption Mechanism

The adsorption mechanism was evaluated by combining experimental characterization with static molecular calculations. FTIR changes after adsorption support interaction of MO with ZIF-67, while electrostatic-potential (ESP) maps identify possible complementary charge regions. These observations are interpreted together with the pH, competing-ion, and regeneration results.

**Figure 12:**
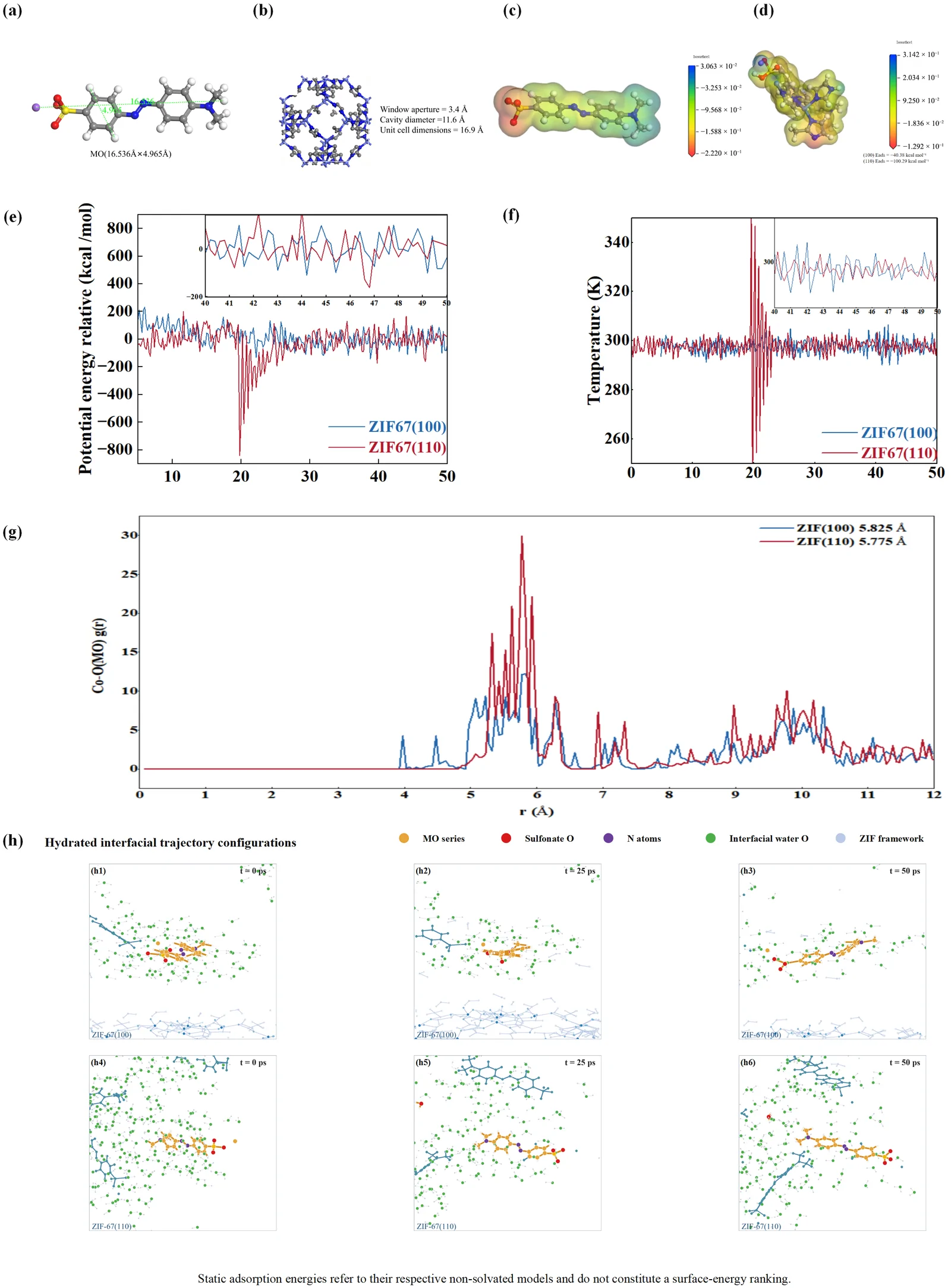
Static characterization and hydrated interfacial dynamics of MO on ZIF-67 facets: (**a**) optimized dimensions of isolated MO, where C, H, N, O, S, and Na atoms are shown in gray, white, blue, red, yellow, and purple, respectively; (**b**) pore-window structure derived from the ZIF-67 crystallographic model, with the Co nodes shown in blue and the imidazolate framework shown in gray; (**c**) DMol^3^-calculated electrostatic-potential surface of isolated MO; (**d**) representative non-solvated MO/ZIF-67 adsorption configuration and facet-specific static adsorption energies; (**e**) potential-energy fluctuations relative to the trajectory mean; (**f**) temperature evolution; (**g**) Co–O(MO) radial distribution functions calculated over 40–50 ps, where the blue and red curves represent ZIF-67(100) and ZIF-67(110), respectively; and (**h**) representative configurations of the hydrated MO/ZIF-67 interfaces: (**h1**) ZIF-67(100) at 0 ps; (**h2**) ZIF-67(100) at 25 ps; (**h3**) ZIF-67(100) at 50 ps; (**h4**) ZIF-67(110) at 0 ps; (**h5**) ZIF-67(110) at 25 ps; and (**h6**) ZIF-67(110) at 50 ps. In panel (**h**), orange, red, purple, green, and light blue represent the MO framework, sulfonate O atoms, N atoms, interfacial-water O atoms, and the ZIF-67 framework, respectively. The dominant RDF maxima occurred at 5.825 Å for ZIF-67(100) and 5.775 Å for ZIF-67(110). The static adsorption energies refer to the respective non-solvated models and should not be interpreted as a surface-energy ranking.

ESP surfaces were calculated for MO and a representative optimized MO/ZIF-67 configuration (Figure 12). Negative potential is concentrated mainly around the sulfonate oxygen atoms of MO, whereas more positive regions occur near the dimethylamino and aromatic moieties. The complementary regions at the optimized interface indicate where electrostatic contacts may be favorable.

The ZIF-67 pore window (~3.4 Å) is smaller than the minimum molecular width of MO (~4.97 Å), indicating that adsorption occurs predominantly at the external surface. The calculated adsorption energies of −40.38 kcal mol^−1^ for (100) and −100.29 kcal mol^−1^ for (110) describe static interactions within the corresponding non-solvated models and should not be interpreted as aqueous adsorption free energies.

Both hydrated systems remained thermally stable during the final analysis interval. Over 40–50 ps, the mean temperatures were 297.68 ± 3.66 K for (100) and 297.63 ± 2.34 K for (110); the corresponding potential-energy standard deviations were 54.92 and 57.92 kcal mol^−1^. Absolute potential energies were not compared because the models contain different numbers of atoms. Within the same interval, the Co–O(MO) RDFs showed dominant maxima at 5.825 Å for (100) and 5.775 Å for (110), with a higher normalized maximum for (110). The absence of a dominant short-range peak does not support persistent direct Co–O coordination. Instead, both trajectories support solvent-separated external-surface association, while the stronger radial ordering on (110) indicates facet-sensitive interfacial organization under the matched coverage conditions. This comparison is based on one 50 ps trajectory per facet and should not be interpreted as a surface-energy ranking or a converged adsorption free-energy calculation. Representative configurations at 0, 25, and 50 ps showed translational and orientational rearrangements of MO while the dye molecules remained associated with the hydrated external ZIF-67 surfaces.

**Figure 13:**
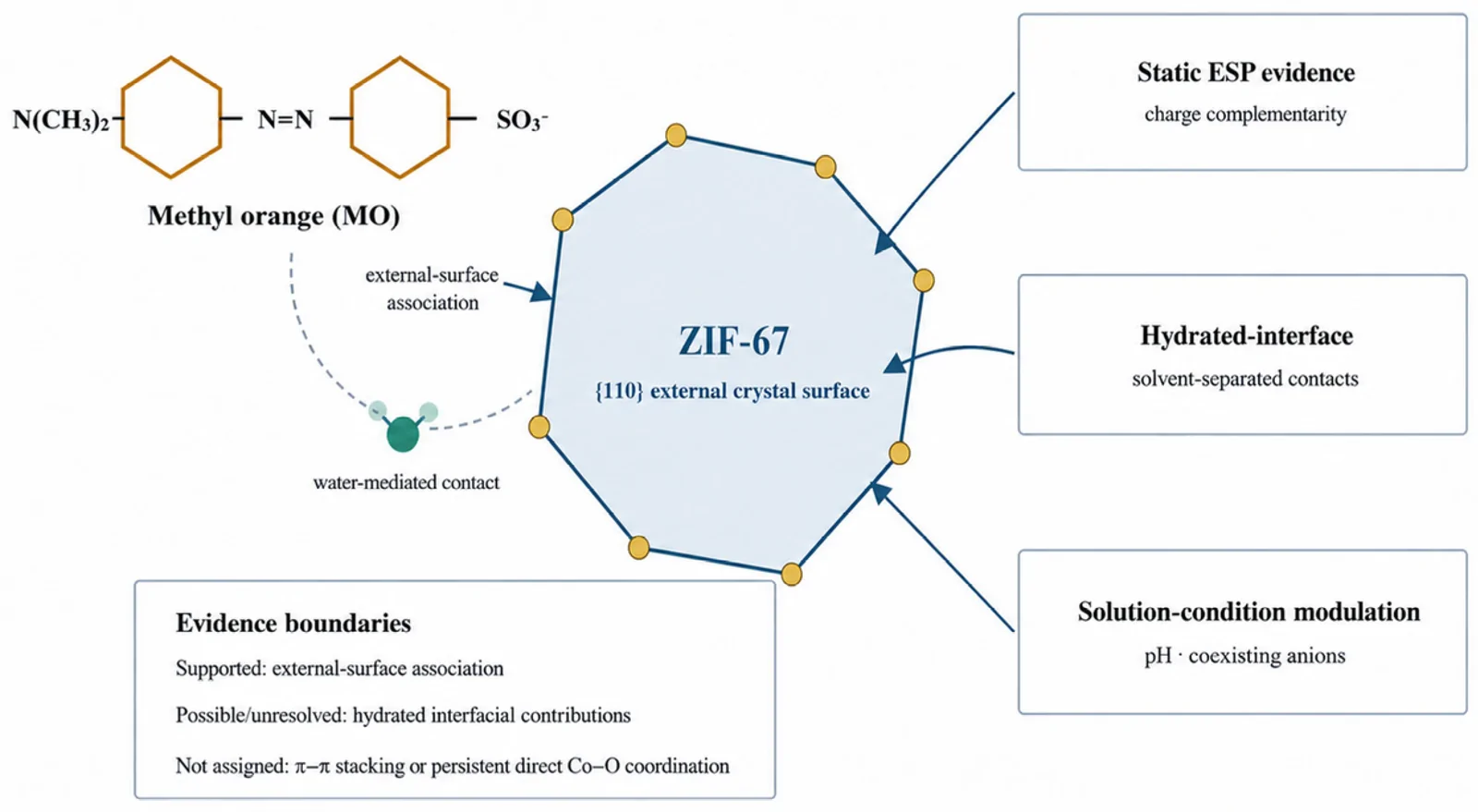
Evidence-bounded model of MO adsorption on ZIF-67. The light-blue polygon represents the external {110} crystal surface of ZIF-67, and the yellow circles denote simplified surface nodes/sites. The green and light-cyan circles schematically represent a water molecule involved in a water-mediated contact. Dark-blue solid arrows connect the principal evidence or condition categories to the interfacial model, whereas gray dashed lines indicate the proposed indirect, water-mediated association pathway between MO and the hydrated ZIF-67 surface.

Figure 13 presents an evidence-supported interfacial adsorption model rather than a uniquely established molecular mechanism. Because the molecular dimensions of methyl orange (MO) exceed the ZIF-67 pore-window size, MO is represented as associating predominantly with the external {110} crystal surface. Static electrostatic-potential analysis identifies complementary charge regions that may favor interfacial association, whereas water molecules and dashed lines illustrate possible solvent-separated, water-mediated contacts. The hydrated MD and Co–O(MO) RDF results do not support persistent direct Co–O coordination, and the individual contributions of electrostatic interactions, dispersion, hydrogen bonding, and solvent reorganization remain unresolved. Solution pH and coexisting anions may further modulate adsorption through changes in surface speciation, competitive interactions, electrostatic screening, and buffering. Because aromatic centroid distances and interplanar-angle distributions were not evaluated, π–π stacking is not assigned.

## 3. Materials and Methods

### 3.1. Reagents and Instruments

Methyl orange, 2-methylimidazole (analytical grade, Shanghai Macklin Biochemical Co., Ltd., Shanghai, China); anhydrous ethanol (analytical grade, Shanghai Macklin Biochemical Technology Co., Ltd.); cobalt nitrate hexahydrate (analytical grade, Shanghai Macklin Biochemical Technology Co., Ltd.); Sodium chloride (NaCl), sodium sulfate (Na_2_SO_4_), and sodium bicarbonate (NaHCO_3_) (analytical grade, Meryer Biochemical Technology Co., Ltd., Shanghai, China) were used as competing anion sources in ion competition experiments. Deionized water was self-prepared distilled water.

Scanning electron microscope (Quattro S with EDS and STEM, Thermo Fisher Scientific Inc., Waltham, MA, USA); X-ray diffractometer (X’Pert MPD PRO PANalytical B.V., Almelo, The Netherlands); Fourier transform infrared spectrometer (Alpha II, Bruker Optics GmbH & Co. KG, Ettlingen, Germany); thermogravimetric analyzer (NETZSCH STA 409 PC/PG, NETZSCH-Gerätebau GmbH, Selb, Germany); UV–visible spectrophotometer (Specord 50 plus, Analytik Jena GmbH+Co. KG, Jena, Germany); Nitrogen adsorption–desorption analyzer (SSA-7000, Beijing Builder Electronic Technology Co., Ltd., Beijing, China); Zeta potential measurement system (STABINO ZETA, Microtrac MRB, Haan, Germany).

### 3.2. Preparation of ZIF-67

**Figure 14:**
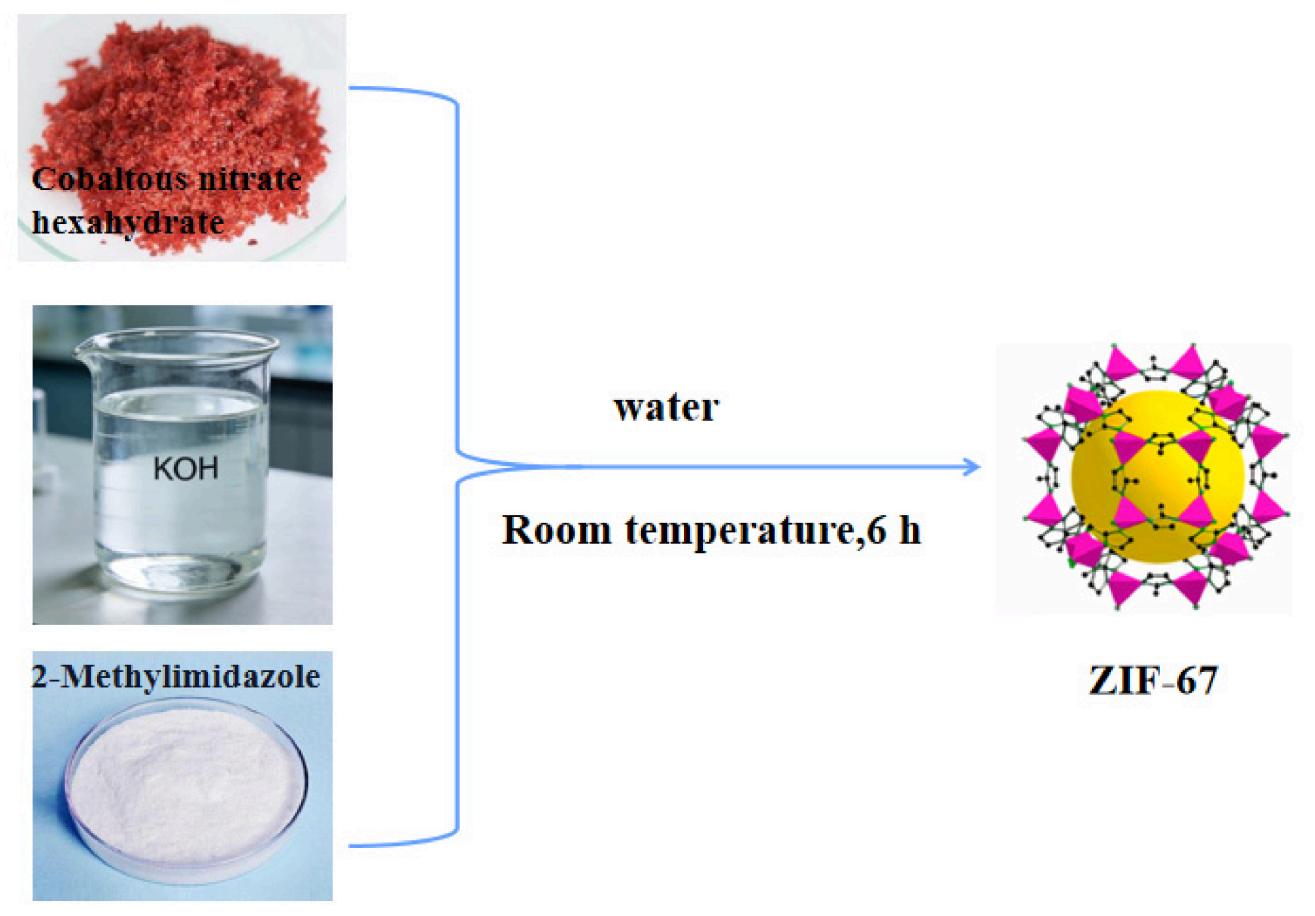
Schematic flow chart for the synthesis of ZIF-67. In this structural model of ZIF-67, pink polyhedra denote Co-centered tetrahedra, black segments represent carbon atoms, green spheres stand for nitrogen atoms, and the yellow sphere schematically shows the internal cavity of the ZIF-67 cage.

ZIF-67 was synthesized using a modified literature method [35]. As shown in Figure 14, 3 mol L^−1^ KOH solution (30 mL) containing 6.0 g of 2MIM was ultrasonicated and stirred for 15 min (solution A). Cobalt nitrate hexahydrate (0.537 g) was dissolved in 30 mL of deionized water (solution B) and added to solution A under stirring. The mixture was stirred at 300 rpm for 6 h at room temperature. The precipitate was washed once with deionized water and three times with ethanol and then dried. This aqueous, room-temperature route avoids organic solvent and elevated temperature during synthesis.

### 3.3. Characterization of ZIF-67 Adsorbent

X-ray diffraction patterns were recorded over a 2θ range of 4–80° using Cu Kα radiation (λ = 0.15418 nm) at an operating voltage of 40 kV, a current of 40 mA, and a scanning rate of 1° min^−1^. Fourier-transform infrared spectra were collected from KBr pellets over the wavenumber range of 4000–400 cm^−1^. For scanning electron microscopy and energy-dispersive X-ray spectroscopy, the powder samples were dispersed in ethanol, deposited onto carbon tape attached to an aluminum stub, dried, and coated with a thin carbon layer to minimize charging. Thermogravimetric analysis was conducted under a nitrogen atmosphere from room temperature to 800 °C at a heating rate of 10 °C min^−1^. Nitrogen adsorption–desorption isotherms were measured at 77 K after the samples had been degassed under nitrogen at 200 °C for 8 h. The specific surface area and pore characteristics were determined from the appropriate regions of the adsorption isotherms. For zeta-potential measurements, the samples were ultrasonically dispersed in deionized water to obtain homogeneous suspensions.

The complementary characterization techniques were used to evaluate the structural and physicochemical properties of ZIF-67. Agreement between the experimental and simulated XRD patterns was used to confirm the crystalline phase and phase purity. In the FTIR spectra, disappearance of the N–H stretching band of free 2-methylimidazole and the appearance of the Co–N vibration supported formation of the ZIF-67 framework. SEM/EDS provided information on particle morphology and elemental composition and distribution, while TGA was used to identify the loss of adsorbed guest molecules and the thermal decomposition of the framework. The type-I N_2_ adsorption–desorption isotherm and the calculated BET surface area were used to characterize the microporous structure. Changes in the zeta potential after MO adsorption provided additional evidence of interactions at the ZIF-67–solution interface.

### 3.4. Adsorption Experiments

All adsorption experiments used simulated wastewater prepared by dissolving MO in deionized water. Solution pH was adjusted with 0.1 mol L^−1^ HCl or NaOH and measured using a calibrated pH meter. In a typical experiment, a specified mass of ZIF-67 was added to 30 mL of MO solution and shaken at 200 rpm. After adsorption, the suspension was centrifuged and the supernatant was analyzed by UV–visible spectroscopy at 465 nm. Isotherm experiments used initial MO concentrations of 10–100 mg L^−1^; kinetic experiments used contact times of 20–180 min. Dosage and pH studies used 20 mg L^−1^ MO, with 15 mg ZIF-67 used for the pH series. Competing-ion experiments used 20–200 mg L^−1^ NaCl, Na_2_SO_4_, or NaHCO_3_ at a fixed initial MO concentration of 20 mg L^−1^. For regeneration, spent ZIF-67 was desorbed in ethanol at 303 K for 180 min, centrifuged at 6000 rpm for 3 min, dried at 60 °C for 24 h, and reused for six cycles.

### 3.5. Computational Methods

**Figure 15:**
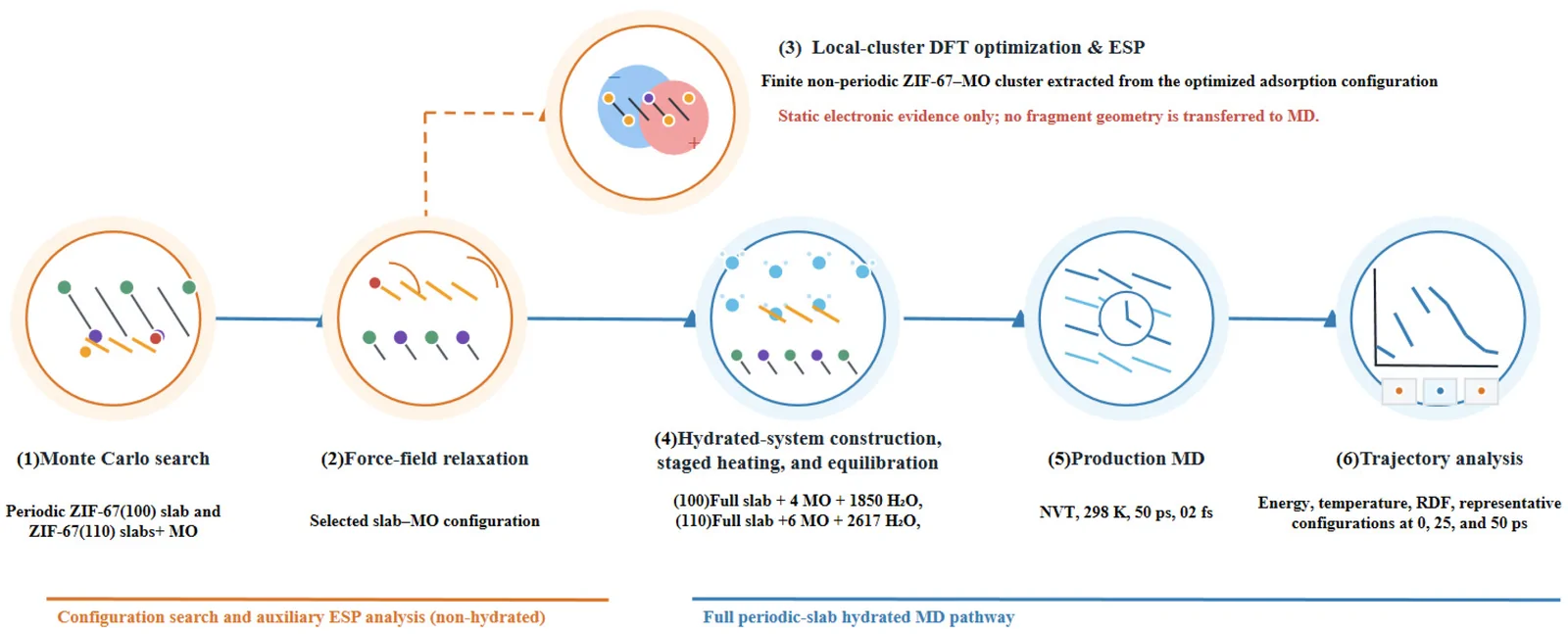
Complete computational workflow used to investigate MO adsorption on ZIF-67(100) and ZIF-67(110). The orange pathway represents non-hydrated adsorption-configuration searching and force-field-based relaxation. The orange dashed arrow denotes an independent auxiliary local-cluster DMol^3^/ESP analysis, which provides static electronic information only; the optimized cluster geometry was not transferred to the subsequent periodic MD simulations. The blue pathway represents the independent construction, staged heating, equilibration, and 50 ps production MD simulations of the hydrated periodic slabs. Potential-energy and temperature profiles were evaluated over the production trajectories, and the conservatively selected 40–50 ps interval was used for RDF and structural analyses. Solid arrows indicate sequential simulation steps. The line segments and colored dots within the circular schematic panels are simplified representations of the ZIF-67 framework, methyl orange (MO) molecules, and water molecules in the different simulation systems.

The complete computational workflow is summarized in Figure 15. This workflow comprises two correlated yet independent computational branches, including non-hydrated adsorption configuration screening and auxiliary electronic structure analysis, as well as periodic hydrated molecular dynamics simulations. The finite cluster adopted for DMol^3^ calculations exclusively acquires static electronic information, without direct integration into the periodic hydrated MD models.

All calculations were performed using Materials Studio 2020. Because the molecular dimensions of MO exceed the pore-window size of ZIF-67, the simulations focused on adsorption at the external crystal surfaces. The original (100) orientation was retained as a reference model, while the morphologically relevant (110) facet was introduced to examine whether the inferred interfacial behavior was orientation-specific. The present comparison was not intended to establish a surface-energy hierarchy or Wulff morphology.

Periodic ZIF-67(100) and ZIF-67(110) slab models were constructed from the crystallographic structure. The crystallographic model was checked for structural disorder, missing atoms, periodicity, and local Co–N coordination before surface construction. The two surfaces were generated using the same surface-termination principle to minimize inconsistencies caused by model construction.

For each surface, low-energy adsorption configurations of methyl orange (MO) were searched under non-hydrated conditions using the Adsorption Locator module in Materials Studio. The resulting configurations were ranked according to their calculated energies, and representative low-energy slab–MO structures were subsequently subjected to force-field-based structural relaxation. This step was used to remove unfavorable atomic contacts and obtain structurally relaxed adsorption configurations before further static analysis.

The static adsorption energy was calculated consistently as the following equations.

(10) $$E_{ads}=E_{slab+MO}-E_{slsb}-E_{MO}$$

where $E_{slab+MO}$, $E_{slsb}$, and $E_{MO}$ are the energies of the relaxed adsorption complex, isolated slab, and isolated MO molecule, respectively. A negative value indicates an energetically favorable adsorption configuration. Because these calculations were performed for non-hydrated static structures, the resulting adsorption energies were used only to compare the selected configurations and were not interpreted as aqueous adsorption free energies.

A finite, non-periodic ZIF-67–MO cluster was extracted from the relaxed adsorption configuration for DMol^3^ calculations. Dangling bonds generated during cluster extraction were terminated with hydrogen atoms. Candidate spin multiplicities were screened, and the electronically reasonable state was retained for subsequent optimization and electrostatic-potential analysis. Separate DMol^3^ calculations were performed for isolated MO and the selected local MO/ZIF-67 clusters. Geometry optimization employed the GGA-BLYP functional, a DNP basis set, and unrestricted spin treatment. Electron density and electrostatic potential were subsequently calculated, and the ESP was mapped onto an electron-density isosurface to assess static charge complementarity between MO and the local ZIF-67 environment.

This local-cluster calculation provided auxiliary static electronic evidence only. Its optimized geometry was not inserted into the hydrated MD system. Instead, the aqueous simulations were independently constructed from the complete periodic slab models, thereby avoiding an artificial transfer from a finite cluster to a periodic interface.

Hydrated periodic models were constructed independently for the two facets. To enable a more meaningful comparison, the MO loading was matched according to accessible surface area rather than by assigning the same number of dye molecules mechanically to both slabs. The water-layer thickness and simulation-cell height were also matched as closely as permitted by the two surface geometries. The periodic (100) and (110) cells had surface areas of 1150.417 and 1626.936 Å^2^, respectively. The lower half of each slab was restrained according to the same geometric rule, whereas the upper framework atoms, MO molecules, and water molecules remained mobile throughout structural relaxation, equilibration, and production sampling.

The final hydrated systems consisted of a complete periodic slab together with corresponding molecules: ZIF-67(100) contains 4 MO molecules and 1850 H_2_O molecules, while ZIF-67(110) contains 6 MO molecules and 2617 H_2_O molecules.

A simulation-cell height of approximately 55.8 Å was used to provide an explicit aqueous region above the slab. The slab constraints and surface-termination treatment were applied consistently to the two models. Before production simulation, each hydrated system underwent structural relaxation, staged heating, and equilibration. This procedure was used to remove unfavorable contacts introduced during packing and to bring the systems gradually to the target temperature of 298 K.

Production MD simulations were performed with the Forcite Dynamics module using the Universal force field and the atomic charges stored in the corresponding input structures. Both facet models were simulated using identical dynamic settings: the NVT ensemble, a target temperature of 298 K, a Nosé thermostat, a time step of 0.2 fs, and a total production time of 50 ps. Each trajectory therefore comprised 250,000 integration steps.

Trajectory frames were saved every 1000 steps, corresponding to a sampling interval of 0.2 ps. Electrostatic interactions were evaluated by Ewald summation with an accuracy of 0.001 kcal mol^−1^, whereas van der Waals interactions were treated by atom-based summation using a cutoff of 12.5 Å. The same random-seed scheme was applied to both simulations, with the random-seed identifier for Seed 1 set to 314159.

Because the simulations employed restrained slabs and fixed simulation cells, the calculated virial pressure was not interpreted as hydrostatic pressure and was not used to compare the two facets. In addition, only one production trajectory was available for each surface; consequently, the simulations provide a descriptive comparison of interfacial behavior rather than a statistically replicated estimate of facet effects.

Potential-energy and temperature profiles were first evaluated over the complete available production trajectories to assess energetic and thermal stability. Both systems exhibited late-stage fluctuations around stable mean values without sustained drift. Accordingly, the final 10 ps interval, 40–50 ps, was conservatively selected as a common production-trajectory analysis window.

The Co–O(MO) radial distribution function was subsequently calculated using trajectory frames extracted exclusively from this predefined interval. Surface Co atoms constituted the first RDF atom set, while the sulfonate oxygen atoms of MO constituted the second set. The same atom definitions, bin width of 0.05 Å, cutoff distance of 12.0 Å, frame interval, and analysis window were used for both facets. Duplicate frames at the boundaries of consecutively generated trajectory segments were removed before analysis, resulting in 51 unique frames for each 40–50 ps trajectory.

Potential energy and temperature were used to assess late-stage stability and select the common analysis interval. Representative snapshots were extracted to illustrate the temporal evolution of the MO–ZIF-67 interface. The RDFs and snapshots were interpreted together to distinguish persistent short-range coordination from solvent-separated or noncovalent interfacial association.

### 3.6. Data Analysis

The following equations were used to calculate the equilibrium adsorption capacity (*q_e_*), adsorption capacity at time *t* (*q_t_*), and removal efficiency (*R*).

(11) $$q_{e}=\frac{(c_{0}-c_{e})V}{m}$$

(12) $$q_{t}=\frac{(c_{0}-c_{t})V}{m}$$

(13) $$R=\frac{c_{0}-c_{e}}{c_{0}}\times 100\%$$

where

*q_e_* and *q_t_* = equilibrium and time-specific adsorption capacities (mg/g), respectively;

*c*_0_, *c_t_*, and *c_e_* = Methyl orange’s initial concentration, concentration at time *t*, and equilibrium concentration (mg/L), respectively;

*V* = volume of methyl orange solution (L);

*m* = mass of ZIF-67 (g);

*R* = equilibrium removal rate (%).

## 4. Conclusions

In this work, ZIF-67 was successfully synthesized via a facile aqueous room-temperature route and applied for the removal of the anionic azo dye methyl orange from aqueous solution. Batch adsorption experiments demonstrated that ZIF-67 possessed favorable adsorption performance toward MO, with the optimal removal efficiency achieved at pH 7.0 and an adsorbent dosage of 15 mg. The adsorption process reached equilibrium within 120 min at 25 °C. Kinetic data conformed to the pseudo-second-order model, while equilibrium isotherms were well-fitted by the Langmuir model. Common coexisting anions including bicarbonate and sulfate exerted inhibitory effects on MO uptake. After six consecutive adsorption–desorption cycles, ZIF-67 still maintained considerable removal performance, illustrating acceptable reusability.

Combining DFT static cluster calculations and explicit-solvent molecular dynamics simulations of representative (100) and (110) crystal facets, we revealed that MO molecules were mainly adsorbed on the external surface of ZIF-67 rather than inside pores. The combined analysis of radial distribution functions and structural snapshots indicated that MO interacts with ZIF-67 predominantly through non-covalent interfacial association, instead of persistent direct Co-O coordination. The two facets exhibited different local radial organization of MO under the modeled conditions.

This study deepens the mechanistic understanding of dye adsorption over ZIF-67 at the molecular level. It provides useful references for designing high-efficiency MOF-based adsorbents targeting organic dye pollutants. Further efforts could focus on multiple simulation replicas, longer simulation time scales, more exposed crystal planes, competing-ion environments, as well as practical real-wastewater verification to promote real-world application of MOF adsorbents.

## Figures and Tables

**Table 1:** Principal FTIR bands and corresponding vibrational assignments of 2MIM and ZIF-67.

Wavenumber (cm^−1^)	Vibrational Assignment
3141	Aromatic C–H stretching of the imidazole ring
2928	Aliphatic C–H stretching of the methyl group
1583	Predominantly C=N stretching of the imidazolate ring
1422, 1305	Imidazolate-ring stretching and deformation modes
1135, 987	C–N stretching coupled with in-plane imidazolate-ring vibrations
754, 690	Out-of-plane imidazolate-ring deformation

**Table 2:** Pseudo-first-order kinetic fitting parameters.

T (°C)	*q*_e,exp_ (mg/g)	*q*_e,cal_ (mg/g)	*k*_1_ (min^−1^)	R^2^
25	36.42	3.85	0.01573	0.5814

**Table 3:** Pseudo-second-order kinetic fitting parameters.

T (°C)	*q*_e,exp_ (mg/g)	*q*_e,cal_ (mg/g)	*k*_2_ (g mg^−1^ min^−1^)	R^2^
25	36.42	36.900369	0.00905	0.99843

**Table 4:** Fitting parameters of adsorption isotherm models.

Model	Parameter	25 °C	35 °C	45 °C
Langmuir Model	q_m_ (mg/g)	156.74	141.43	143.18
Langmuir Model	K_L_ (L/mg)	0.191	0.159	0.1389
Langmuir Model	R^2^	0.996	0.969	0.970
Freundlich Model	n	2.312	2.34	2.38
Freundlich Model	K_F_ (mg/g)	354.21	26.48	25.31
Freundlich Model	R^2^	0.846	0.958	0.9534

**Table 5:** Comparison of adsorption capacity for MO removal.

Adsorbent	qmax (mg/g)	Conditions	BET (m^2^/g)	Reference
ZIF-67 (this work)	156.74	25 °C, pH 7	1842.31	This work
Orange and lemon peel-derived activated carbon	38	30 °C, pH 2	168.29	[52]
Zeolite Y	20.62	25 °C, pH 8	445.36	[53]
Amino-ethyl carboxymethyl cellulose crosslinked ampholyte hydrogel	1.8	25 °C, pH 7	/	[54]
160-MIL-101	444.3	25 °C	3021	[55]
MOR-1-HA	859	25 °C, pH 7	1182	[56]
MOF-235	477	25 °C	/	[57]
MIL-100(Fe)	1045.2	30 °C, pH 5	/	[58]
U-B-HCl	528.6	25 °C	1538.2	[59]
Chitosan	8.867	60 °C, pH 4	/	[60]
Ch/Su Schiff base	10.00	60 °C, pH 4	/	[60]
Ch/Mp Schiff base	7.20	60 °C, pH 4	/	[60]
ZIF-8@0.5GO	82.78	25 °C, pH 5.5	286.22	[61]
MIL-101 (1.6, 2.1)	114	25 °C, pH 3	3873	[62]

**Table 6:** Thermodynamics adsorption parameters of MO onto ZIF-67.

Samples	T (°C)	ΔG (kJ/mol)	ΔH (kJ/mol)	ΔS (J/(mol·K))
ZIF-67	25	−23.73	−8.67	+50.5
	35	−24.29		
	45	−24.74		

## Data Availability

The original contributions presented in this study are included in the article. Further inquiries can be directed to the corresponding author.
